# Foundation model-powered deep learning of endometrial histology for predicting the cumulative live birth of an *in vitro* fertilization cycle

**DOI:** 10.1371/journal.pdig.0001744

**Published:** 2026-09-28

**Authors:** Nianbo Xu, Andy Chun Hang Chen, Hanzhang Ruan, Donglin Yang, Renjie Liao, Xiaojuan Qi, Sze Wan Fong, Dandan Cao, Lu Yu, Yuanhua Huang, William Shu Biu Yeung, Ernest Hung Yu Ng, Yin Lau Lee

**Affiliations:** 1 Department of Obstetrics and Gynaecology, School of Clinical Medicine, Li Ka Shing Faculty of Medicine, The University of Hong Kong, Hong Kong SAR, China; 2 InnoHK Centre for Translational Stem Cell Biology, The Hong Kong Science and Technology Park, Hong Kong SAR, China; 3 Shenzhen Key Laboratory of Fertility Regulation, The University of Hong Kong Shenzhen Hospital, Shenzhen, China; 4 Department of Electrical and Electronic Engineering, The University of Hong Kong; 5 Department of Electrical and Computer Engineering, University of British Columbia, Vancouver, Canada; 6 Vector Institute for Artificial Intelligence, Toronto, Canada; 7 Canadian Institute for Advanced Research (CIFAR) Artificial Intelligence Chair, Toronto, Canada; 8 School of Biomedical Sciences, The University of Hong Kong, Hong Kong SAR, China; Tianjin Medical University General Hospital, CHINA

## Abstract

Objective and reproducible assessment of endometrial receptivity is essential for optimizing *in vitro* fertilization (IVF) success, yet traditional histological dating suffers from observer variability. This study investigated whether deep learning of hematoxylin and eosin histology images could support cumulative live birth prediction in IVF. An end-to-end ResNet-18 was compared with a UNI2-h-based pipeline, using UNI2-h as a frozen feature extractor. Ten-fold cross-validation ensembles were developed from natural-cycle endometrial biopsies and evaluated in an internal held-out cohort with live birth outcomes. Additional phase-based evaluation was performed, which tested the performance in distinguishing LH + 7 versus non-LH + 7 phases. A luminal epithelium (LE)-focused design was also assessed to examine whether concentrating on maternal-embryo interface could improve fertility-oriented learning. The whole-slide ResNet-18 model performed well in internal outcome-based testing but near random in external phase-based testing. In contrast, the UNI2-h model with mean pooling and a multilayer perceptron classifier showed less divergent performance, with ensembled AUROCs of 0.74 ± 0.03 in internal outcome-based evaluation, and 0.90 ± 0.05 in external phase-based testing. Despite a smaller training set, LE-focused models retained comparable performance. In internal outcome-based testing, LE-focused ResNet-18 and UNI2-h models achieved AUROCs of 0.71 ± 0.03 and 0.74 ± 0.09 respectively. External phase-based testing yielded AUROCs of 0.80 ± 0.16 for ResNet-18, and 0.84 ± 0.06 for UNI2-h. Grad-CAM review of LE-focused ResNet-18 models showed attention commonly on LE-alone or mixed with adjacent stroma. In multimodal analyses, integrated models incorporating histology significantly outperformed the clinical metadata-only model. Integrated model’s feature weighting showed dominant histology outputs, smaller contributions from estradiol and maternal age, and negligible contributions from progesterone, endometrial thickness, and BMI. These findings support histology-based AI for fertility-oriented endometrial assessment and highlight biologically informed design and repurposed foundation models as promising bases for clinically meaningful prediction.

## Author summary

Clinicians still lack an objective way to assess whether the endometrium is in an optimal state to support a successful pregnancy. Traditional histological assessment relies on pathologists’ interpretations and vary among observers, while newer molecular tests remain costly and uncertain reliability. In this study, we explored whether artificial intelligence (AI) could extract useful fertility-related information from routine endometrial histology slides. We compared a conventional training approach with a more modern strategy that repurposed an existing AI model with pre-existing histology knowledge. Two testing cohorts, including an internal cohort based on live-birth outcomes and an external cohort evaluated on luteal-phase classification were included. The repurposed approach showed more consistent performance between the two testing cohorts, while the conventional approach fell to near-random in the external phase-based cohort. We also tested whether focusing on endometrial luminal epithelium, where the embryo first makes contact, could improve prediction. Surprisingly, luminal epithelium-focused models performed comparably to the whole-slide approach despite using far fewer training images. These findings support AI-based histology analysis as a promising approach that warrants further refinement for more objective endometrial assessments. Combining foundation models with biologically informed design could provide a useful direction for future fertility-related diagnostics.

## Introduction

The global birth rate remains low as many countries report their birth rate lower than the replacement level of 2.1 births per woman. The current cumulative live birth rate per cycle remained at ~30–40% only [1] despite advancements in *in vitro* fertilization (IVF). Poor embryo quality is often cited as the primary factor in infertility, but the probability of a live birth per a single euploid embryo transfer stays at approximately 60% [2,3], suggesting that maternal factors beyond embryo euploidy contribute to the outcomes. Consequently, a large proportion of patients require multiple transfer cycles, which can be particularly challenging for patients with a limited number of good quality embryos. Repeated failures are also significantly correlated with a higher risk of anxiety and depression even after accounting for socioeconomic confounding factors [4,5].

Optimal endometrial preparation is essential for the establishment of a successful pregnancy [6]. Yet, clinicians still lack an accurate and low cost method to assess endometrial receptivity and predict live birth outcomes [7]. Conventional non-invasive markers, such as ultrasound-based endometrial thickness and vascularization fail to serve as reliable predictive tools because of conflicting results reported [8–10]. The Noyes criteria, which provided descriptive morphological standards to date endometrial samples, has long been the “gold standard” of endometrial histological dating since its introduction in 1950 [11]. However, the technique is labor intensive and exhibits significant observer variability, compromising its reliability [12,13]. Advancements in understanding the molecular signatures of the window of implantation (WOI) have provided hopes of novel assays for endometrial dating. Up-to-date, the true clinical value of currently available molecular assays, like the Endometrial Receptivity Analysis (ERA), remains controversial due to unproven benefits in predicting IVF success [14–16]. Thus, there is a critical need for an objective and low-cost method to evaluate endometrial receptivity.

Since the introduction of the initial whole slide image (WSI) scanner in the 1990s, computational pathology has evolved from simple feature extraction to advanced deep learning algorithms capable of interpreting complex high-dimensional features [17]. Deep learning models are commonly developed either by full training from scratch or by adapting pretrained foundation models. The full training approach, typically utilizing Convolutional Neural Networks (CNNs), enables task-specific learning but is inherently data-demanding and sensitive to institutional biases such as imaging and staining variations [18,19]. Consequently, the field is increasingly shifting toward foundation models as a more robust alternative [20–22]. Unlike conventional approaches requiring large task-specific datasets, foundation models such as UNI [21] and Virchow [22] are pretrained on massive histology archives, making the utilization of complex Vision Transformer (ViT) backbones practically feasible. The increasing availability of these foundation models represents a critical methodological advance to make the development of task-specific pathological deep learning models easier, especially when sample sizes are restricted [23].

The application of deep learning based artificial intelligence (AI) model in healthcare is a fast-growing field with immense potential [24]. While AI has been widely tested for its application in optimizing stimulation protocols and embryo selection, its use in predicting endometrial receptivity remains limited. By training a ResNet-18 model from scratch, a widely adopted efficient architecture for natural and medical image analysis [25,26], to differentiate endometrial samples based on fertility outcomes, we reported the earliest application of deep learning in endometrial receptivity [27]. While the reported models achieved satisfactory live birth prediction with an accuracy of 75%, the wider applicability of the model which was derived exclusively from patients undergoing Hormone Replacement Therapy (HRT) was constrained. Endometrium in HRT cycles exhibits distinct histological profiles compared to those in natural cycles [28,29]. Therefore, the generalizability of such a model to natural cycles remains unverified. Furthermore, a significant knowledge gap remains regarding the transferability of modern foundation models to the endometrial function-oriented tasks. Many widely used histopathology foundation models have been developed and extensively benchmarked in cancer-heavy settings [20–22]. It is currently unknown whether these cancer-specific extractors can effectively characterize the subtle, non-malignant morphological changes associated with endometrial receptivity in healthy tissues.

In this study, we aimed to first evaluate the potential transferability of the HRT-trained ResNet-18 model for assessing the performance using WSIs obtained in natural cycles. We also evaluated whether the histopathology pretrained UNI2-h foundation model could provide a simpler, more robust starting point for digital pathology-based fertility outcome prediction. As luminal epithelium (LE) of endometrium is the first point of contact between maternal tissue and the implanting embryo, we also explored an LE-focused modelling approach specific to the fertility-oriented predictive goal.

## Materials and methods

### Ethical approval

The study protocol was approved by the Institutional Review Boards of the University of Hong Kong/Hospital Authority Hong Kong West Cluster (IRB: UW 25–573) and the Ethics Committee of the University of Hong Kong-Shenzhen Hospital (IRB reference number [2026]087).

### Study cohorts and sample collection

Archived anonymized paraffin-embedded tissues with known pregnancy outcomes from a previous study [30] were used in this study. IVF patients who had regular cycles and underwent their first IVF cycles at the Centre of Assisted Reproduction and Embryology, The University of Hong Kong - Queen Mary Hospital and the Assisted Reproduction Centre of Kwong Wah Hospital from 2017 to 2021 were included in this study. They were asked to donate endometrial biopsy samples in their natural cycles. Individuals with [1] an abnormal uterine cavity as determined by saline infusion sonogram or hysteroscopy; [2] untreated uterine pathologies such as endometrial polyps or fibroids; [3] untreated hydrosalpinx; and [4] use of donor oocytes were excluded. Pipelle samplers (CCD Laboratories, Paris, France) were used to obtain the endometrial biopsy at 7 days after a luteinizing hormone (LH) surge (LH + 7). Biopsied samples were fixed and embedded in paraffin blocks. From each block, 4 μm tissue sections were prepared for hematoxylin and eosin (H&E) staining, and the stained slides were digitized as high resolution WSIs at 40 × magnification (approximately 0.220 µm per pixel, or mpp) using a Hamamatsu NanoZoomer S210 slide scanner (Hamamatsu Photonics K.K., Shizuoka Prefecture, Japan). The digitalized WSI files were stored natively in NDPI files first before transformed for further formatting change and downstream processing. In total, 183 internal samples were included in this study, of which 152 samples were assigned for training and validation purposes, and another 31 samples were assigned as an internal held-out test set. Essential metadata and clinical characteristics were summarized in S1 Table.

We also included an external phase-based cohort consisting of 21 fertile patients WSIs collected at natural cycle from the University of Hong Kong-Shenzhen Hospital. The patient sample condition and tissue collection details were described previously [31]. All donors had achieved a healthy live birth within two years of collection either via spontaneous pregnancy or a preimplantation genetic testing (PGT)-validated euploid embryo transfer. The collected samples were biopsied at five different endometrial menstrual phases, including LH + 3 (n = 3), LH + 5 (n = 3), LH + 7 (n = 9), LH + 9 (n = 3), and LH + 11 (n = 3). Paraffin block embedding and H&E staining were conducted at the University of Hong Kong-Shenzhen Hospital. The slides were scanned at 20 × scanning resolution (approximately equivalent to 0.440 mpp) using a KF-FL-020 WSI scanner (KFBIO, Zhejiang, China). The resulting digitalized WSI files were stored natively in KFB file format before transformed for further processing.

### Ovarian stimulation and embryo transfer

Clinical management and IVF procedures were conducted according to the centers’ standard operating procedures as described earlier [30]. Briefly, ovarian stimulation using a gonadotropin-releasing hormone antagonist protocol was used and the starting dosage of FSH depended on the antral follicle count and body mass index of the patients. Final oocyte maturation was triggered with recombinant human chorionic gonadotropin (hCG), followed by oocyte retrieval 34–36 hours later. Embryos were cultured to reach cleavage or blastocyst stage and eventually selected for the subsequent fresh or frozen embryo transfer (FET) cycles. The blastocysts were graded based on the Gardner scoring system [32].

### Outcome data collection

The primary outcome was the cumulative live birth, defined as a live birth occurring after 22 completed weeks of gestation [33] from a stimulated IVF cycle and its subsequent FET cycles within 6 months after start of ovarian stimulation. The outcomes were assigned as either live birth or unsuccessful outcomes. This binary classification was utilized as the ground truths for training and assessing the performance of the developed models in the internal dataset. Ultimately, among the 152 samples utilized for training purposes, 79 were labeled as unsuccessful and 73 were labeled as live birth. The 31 internal held-out testing cohort consisted of 20 unsuccessful and 11 live birth samples.

As the external phase-based cohort comprised fertile donors who did not undergo embryo transfer, direct cumulative live birth outcome labels were unavailable. Therefore, the labels of external phase-based testing cohort were inferred by the timing of the biopsy relative to the LH surge. LH + 7 was considered as receptive, as it is widely accepted to be the optimal timepoint for embryo transfer [31,34]. Our previous time-series single-cell data further supported marked transcriptomic transitions before and after LH + 7, with stromal and glandular epithelial cells showing major changes from LH + 5 to LH + 7, and epithelial cells exhibiting a further sharp transition at late LH + 9 [31]. Thus, a non-identical but similar phase-based binary label was assigned, with LH + 7 samples (n = 9) representing the live birth outcome samples, and pre-receptive (LH + 3, + 5) and post-receptive (LH + 9, + 11) samples collectively representing unsuccessful outcome samples (n = 12). This external testing design therefore evaluated a biologically relevant but non-equivalent target, focusing on distinguishing receptive-phase LH + 7 endometrium from non-LH + 7 samples, rather than directly validating cumulative live birth prediction.

### Histology-based model selection and training parameter settings

This study utilized two separate deep learning architectures, the ImageNet 1K-pretrained ResNet-18 [26] and the histopathology pretrained UNI2-h [21], to achieve the same goal of developing a binary classification model to differentiate endometrial H&E WSIs based on their live birth outcome potentials. All the models generated were trained on a remote Linux server implemented with three available NVIDIA A100 Tensor Core GPUs, except for the HRT-trained ResNet-18 model (HRT-model), which was trained with details as described previously [27].

For all models, an optimal threshold was determined independently for each cross-validation fold by maximizing Youden’s J statistic on the validation cohort (i.e., predicted output higher than the calculated threshold interpreted as live birth; values lower than the threshold interpreted as unsuccessful). Adapting the cross-validation framework from previous studies, we implemented a 10-fold cross-validation strategy to construct an ensemble model, an approach demonstrated to enhance robustness and generalizability compared to individual models [27,35]. In brief, an ensemble was formed by selecting the best-performing model from each fold. The predicted outcomes were averaged with equal weighting to produce a final predicted probability (soft voting), and optimal thresholds were similarly averaged to establish a global threshold.

The adopted CNN-based ImageNet 1K-pretrained ResNet-18 was maintained largely consistent with previous studies by utilizing a linear classifier head for binary classification [27]. Since this model is designed to operate as a patch-level classifier, slide-level predictions were generated by calculating the mean of the predicted probability scores across all patches extracted from a single WSI (Fig 1). To better encounter overfitting issues with the limited sample size, the architecture was modified to include a dropout layer after global average pooling (GAP). Furthermore, conventional data augmentation techniques were applied during training, including random horizontal and vertical flips, rotation, and color jitter. The models were trained using the AdamW optimizer with a cosine decay learning-rate scheduler and a warmup period to prevent destabilizing model weights. Detailed hyperparameter settings, including learning rates, batch sizes, and weight decay values, are summarized in S2 Table.

**Fig 1:**
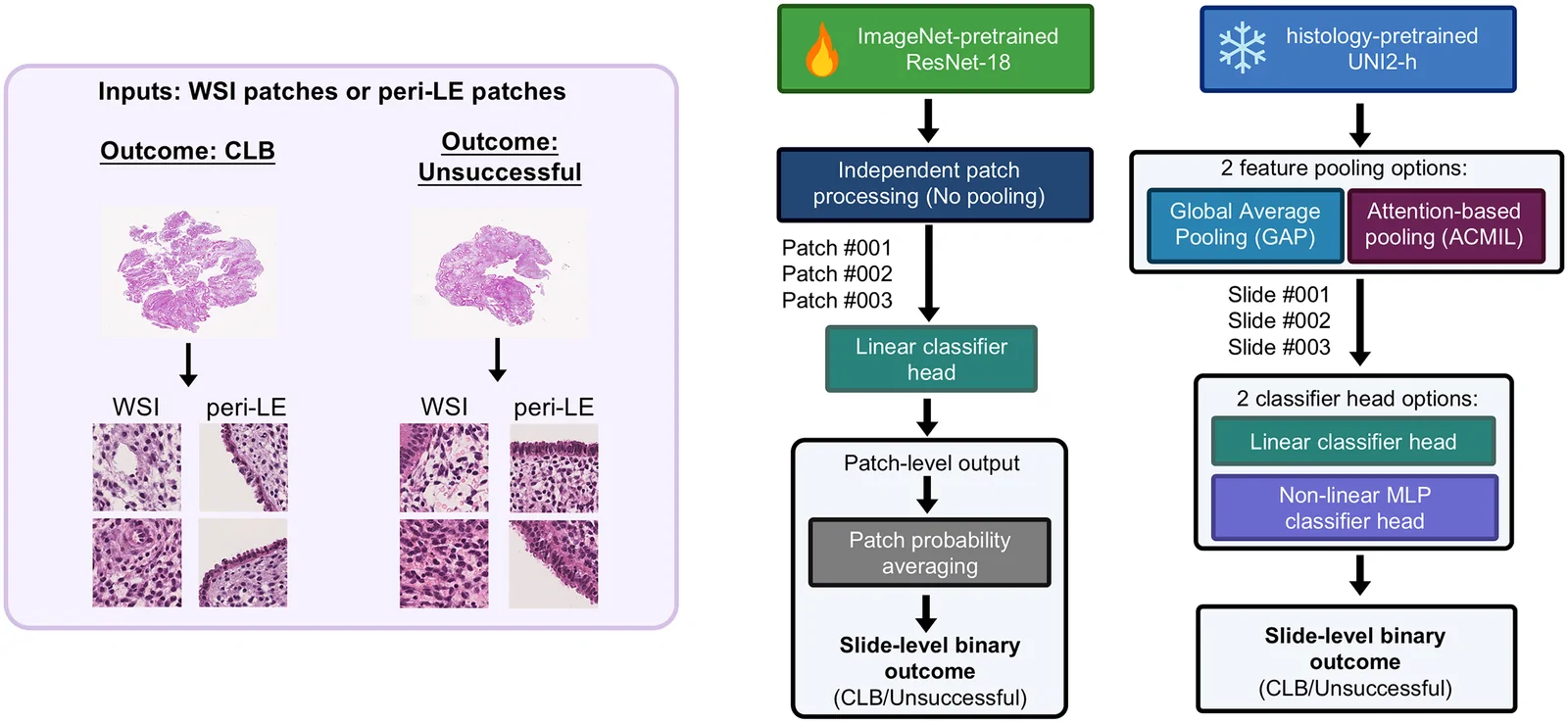
Overview of deep learning pipelines for endometrial fertility outcome prediction. The workflow utilizes input patches derived either from whole slide images (WSIs) or manually annotated LE/peri-LE regions. The samples included two outcomes, either a cumulative live birth (CLB) or unsuccessful outcome. Two distinct modeling strategies were implemented. One includes an ImageNet 1K-pretrained ResNet-18 model trained end-to-end as a patch-level classifier, with patch-level probability scores processed independently and averaged in the end to generate a final slide-level binary prediction (CLB vs. Unsuccessful). The second strategy utilizes a histopathology pre-trained UNI2-h foundation model as a feature extractor. Extracted patch features were aggregated via either a global averaged pooling (GAP) or an attention-based pooling strategy (ACMIL). The aggregated slide-level representation was classified using either a linear classifier head or a non-linear MLP head to predict the slide-level binary outcome. Figure partially created in BioRender [https://biorender.com/f125abi].

The ViT-H/14-based histopathology pretrained UNI2-h foundation model was utilized as a feature extractor to extract high dimensional features from image patches [21]. Considering the size of the UNI2-h model at 681M parameters and the relatively limited abundance of our available sample set, the model’s weights were kept completely frozen during the study. To map these patch-level features to slide-level predictions, two distinctive feature aggregation strategies were implemented (Fig 1). One configuration utilized a GAP mechanism, in which the patch-level features extracted from a single slide were averaged to form a slide-level bag. In parallel, an Attention-Challenging Multiple Instance Learning (ACMIL) mechanism [36] was adapted. ACMIL replaced the equal averaging with a learnable attention network which differentially weight patches based on their discriminative relevance. Averaged or weighted feature outputs from the aggregation mechanisms were eventually passed on to either a conventional linear or a non-linear Multi-Layer Perceptron (MLP) classifier head for the final binary prediction. The MLP variant comprised two hidden layers with 768 and 384 dimensions, and provided a non-linear projection to adapt the UNI2-h features to the current specific endometrial task. For all the UNI2-h extracted feature-based training, one NVIDIA A100 GPU was used. The hyperparameter settings were kept largely consistent across the WSI models, except for necessary adaptations to fit specific model’s input expectations. Detailed hyperparameter settings are shown in S2 Table.

For the LE-focused models specifically trained with patches containing LE and peri-LE stroma regions, the architectures and major parameter settings were kept consistent with the WSI approaches (S2 Table). Specifically, the ResNet-18 LE models were trained using the ImageNet 1K-pretrained backbone combined with a linear classifier head, while the UNI2-h LE models utilized the frozen feature extractor combined with an MLP head.

### Patch image extraction and preprocessing

Direct processing of WSIs in full resolution during training remains impractical due to their typical gigapixel-scale dimensions. Therefore, all WSIs were cropped into smaller patch images in order to facilitate efficient training. Although the maximum native scanning resolution differed between the two cohorts, with internal samples scanned at 40× and external samples scanned at 20 × , all subsequent training and analyses were standardized to patches generated at 20 × resolution (0.440 mpp) for minimizing technical variations. An exception was the internal dataset used for testing the pre-developed HRT model, which utilized an unchanged 40× (0.220 mpp) resolution at 4832 × 6864 pixels to match its specific input settings as described previously [27].

For all WSI models, automated segmentation was performed using the Trident toolkit [37], and only patches with at least 35% of tissue were included. The size of the resulting patch datasets varied slightly depending on the expected input pixel dimension of specific model. For the pipeline involving the ResNet-18 WSI models, all involved patches were extracted at 20 × resolution with a dimension of 224 × 224 pixels. This dataset comprised a training set of 152 patients (92,738 patches), an internal testing set of 31 patients (22,361 patches), and an external phase-based testing set of 21 patients (33,011 patches). In parallel, for pipeline involving the UNI2-h WSI models, all involved patches were extracted at 20 × resolution with dimensions of 256 × 256 pixels. This dataset comprised a training set of 152 patients (85,827 patches), an internal testing set of 31 patients (17,713 patches), and an external phase-based testing set of 21 patients (31,873 patches).

In contrast, the LE-focused workflow relied on manual annotation. Approximate LE-containing region annotations were drawn using QuPath, a WSI viewing and processing tool, followed by automatic generation of fixed-size squares to cover the annotated Regions of Interest (ROIs). These squares were manually adjusted to maximize LE coverage while minimizing the inclusion of glandular structures and were subsequently extracted using a custom Groovy script. Unlike the WSI approach which covers the whole tissue, conducting separate rounds of manual annotation for each pipeline could introduce bias, leading to inconsistencies in areas such as ROI centering and stromal proportion between the two model inputs. Hence, a single set of ‘base’ patches was generated first to ensure consistent anatomical coverage across the two pipelines. Specifically, a series of base patches was first extracted at 260 × 260 pixels (20 × resolution) from the internal and external cohort, and subsequently center-cropped to match the input dimensions of each architecture (224 × 224 pixels for ResNet-18-based and 256 × 256 pixels for UNI2-h feature-based). Consequently, the patch counts were identical for both pipelines: a training set of 124 patients (3,552 patches), an internal testing set of 24 patients (657 patches), and an external phase-based testing set of 18 patients (783 patches).

### Visualization and quantification of model attention regions

The gradient-weighted class activation mapping (Grad-CAM) approach [38] was used to visualize the attention regions on the original patch images of LE models. All patch images from the internal testing cohort (657 images) were utilized to generate the attention heatmaps. In these heatmaps, warmer (red) areas highlighted regions the model deemed more important for its prediction, while cooler (blue) areas had weaker contributions. Semi-quantitative analysis of the Grad-CAM-generated heatmaps was performed by manually evaluating the most prominent high-attention area within the LE-containing patches using the internal testing cohort. Attention categories were assigned according to the predominant localization of the highlighted region. Considering that the patches primarily consisted of LE, peri-LE stroma, and lumen, which were generally easily distinguishable on H&E images, Grad-CAM attention was prespecified into four broad categories comprising LE-predominant, mixed LE and peri-LE stroma, peri-LE stroma-predominant, and patterns on unclassified regions such as lumen and potential artifacts. Other cell types potentially associated with fertility outcomes, such as endothelial and immune cells, were not classified separately because they could not be identified consistently across patches and Grad-CAM provided only approximate spatial localization. Formal categorization was performed independently using anonymized patch identifiers and images, without access to model predictions, prediction probabilities, prediction correctness, patient outcomes, or other clinical information. Based on the average AUROCs across the internal and external sets, three best-performing models from the 10-fold ensembles were selected to visualize for their attention tendencies. Semi-quantitative summaries of attention category distributions were calculated separately for each model and reported as mean proportions with standard deviations. Native ACMIL attention weights from the ACMIL-based WSI pipelines were also directly visualized on WSIs for qualitative inspection of any potentially unique attention patterns.

### Multimodal analysis for clinical metadata integration

To evaluate the potential of multimodal analysis, logistic regression models were constructed using predicted probabilities from the WSI and LE histology models, with or without maternal age (Age), body mass index (BMI), endometrial thickness (EnTh), and biopsy-day serum estradiol (E2) and progesterone (P4) levels within the internal test set. EnTh was determined via transvaginal ultrasound prior to the biopsy for sample collection. Five logistic regression settings were evaluated including: a histology-only model integrating WSI and LE models’ probabilities; an integrative seven-factor histology-clinical model additionally incorporating Age, BMI, EnTh, E2 and P4; and three clinical metadata-only models incorporating Age, BMI and EnTh; E2 and P4; or all five clinical variables. To maximize training efficiency while preventing data leakage, we leveraged the 10-fold cross-validation framework of the histology models to generate out-of-fold predictions for the entire training cohort. These out-of-fold probabilities served as the input features for training the logistic regression models. Multimodal analyses containing histology-based probabilities as inputs were restricted to samples with both WSI and LE model outputs. To examine feature contribution patterns in the most integrative seven-factor model, standardized coefficients weights were calculated by multiplying each raw coefficient by the feature’s standard deviation, allowing the presented coefficients weights to be directly comparable. SHapley Additive exPlanations (SHAP) summary plots were additionally generated for the internal cohorts to summarize the direction and distribution of each feature’s contribution to the predictions.

### Computational software packages

The ImageNet 1K-pretrained ResNet-18 (https://download.pytorch.org/models/resnet18-5c106cde.pth) was trained and the histopathology pretrained UNI2-h foundation model (https://huggingface.co/MahmoodLab/UNI2-h) was utilized directly in this study. The UNI2-h model weights were accessed via application through an institutional email-linked HuggingFace account as requested. Training of models within the study and Grad-CAM visualization largely relied on functions and tools available from the PyTorch machine learning library on Python (PyTorch version = 2.4.1; Python version = 3.10.0) [39]. Logistic regression models for multimodal analysis were implemented using scikit-learn (version = 1.7.1), and SHAP-based model interpretation was performed using the SHAP package (version = 0.49.1). QuPath (version = 0.5.1) was the primary WSI inspection software. Trident (version 0.2.0) is a toolkit developed by the same group that created the UNI2-h [37]. The toolkit was used in all our reported ResNet-18 and UNI2-h-related patch preparation tasks, with Tiff patch images generated for the ResNet-18 workflow and patch-level feature vectors extracted for the UNI2-h-related workflow. Patch image generation for the LE-focused model was performed using QuPath and a Groovy script. DeLong tests were implemented following the fast DeLong algorithm described by Sun and Xu [40].

### Model performance evaluation and reported metrics

The performances of the developed 10-fold ensemble models were evaluated based on three common machine learning performance metrics, including accuracy, balanced accuracy, and area under the receiver operating characteristic curve (AUROC). To monitor training stability and convergence among different architectures and classifier heads, performance trajectories were tracked across all 10 folds and reported as the mean of available folds per epoch to account for varying early-stopping points. To address the potential misleading interpretation of accuracy considering the study’s test sample sets were not in 1:1 balanced ratio, balanced accuracy was utilized to define best epochs during training and validation sessions.

Reported models were compared in two major settings using DeLong test, including with a chance-level random classifier with AUROC = 0.5, and paired model-to-model comparisons. Comparison between the WSI and the LE model was restricted to samples with predictions from both pipelines to ensure paired testing. Reported P values were adjusted using the Benjamini-Hochberg procedure within each cohort and comparison group, with statistical significance defined as an adjusted P < 0.05. To supplement AUROC-based discrimination and threshold-dependent classification metrics, model calibration was assessed using calibration curves and Brier scores. Each model was analyzed separately in the internal cumulative-live-birth testing cohort and the external phase-based testing cohort.

## Results

### Limited transferability of the HRT model to natural cycle WSIs

To evaluate the potential transferability of the existing deep learning model, we first assessed the performance of the reported HRT model [27] using the natural cycle LH + 7 WSIs archived in our centers. The input settings of all the patches, including patch resolution, dimensions, and cropping procedures, were prepared to match its published HRT model. Despite the currently applied input-level standardization, the model yielded an AUROC of 0.55 ± 0.08 and a balanced accuracy of 55.85 ± 1.37%, indicating a limited discriminative capability in this natural cycle cohort (S1 Fig; S3 Table).

### Natural cycle endometrial WSI-trained new models for live birth outcome prediction

We next explored new models using a new WSI workflow for the natural cycle dataset (Fig 2A). We first retrained a new model based on the same ResNet-18 architecture by incorporating adjusted normalization parameters and augmentation strategies tailored to the new dataset. To investigate whether feature representations learned from large pan-cancer datasets could be transferable to a non-cancerous endometrium, live birth-oriented task, we also implemented the UNI2-h foundation model as a frozen feature extractor, evaluating its performance across different aggregation strategies (GAP vs. ACMIL) and classifier heads (linear vs. MLP) with detailed performance metrics summarized in Table 1.

**Fig 2:**
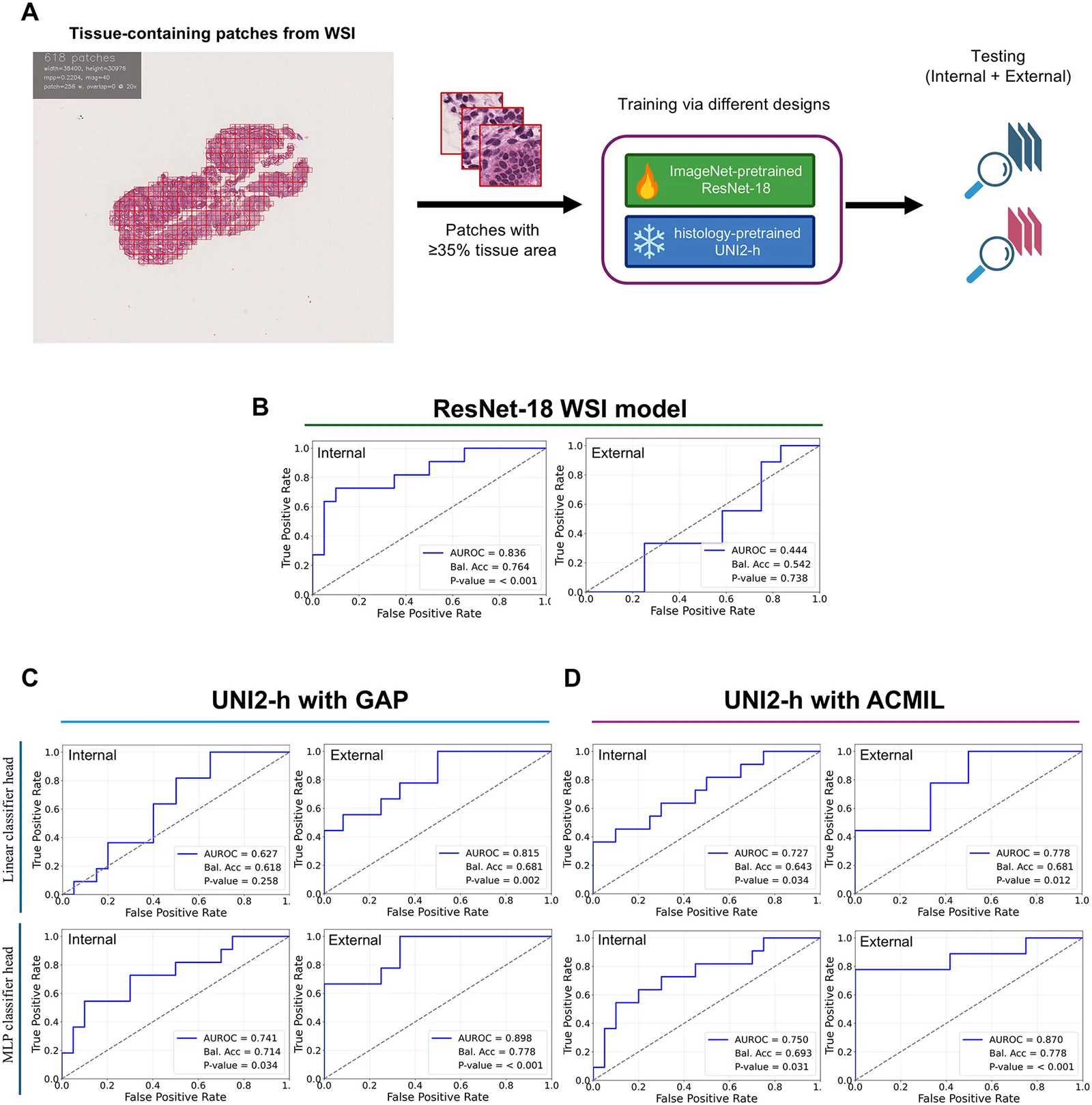
WSI endometrial live birth outcome prediction models. (**A**) Schematic diagram showing the training workflow of the WSI models developed in this study. (**B-D**) ROC curves illustrating the performances of internal and external phase-based test sets of ResNet-18 model (B), UNI2-h GAP-Linear and GAP-MLP models (C) and UNI2-h ACMIL-Linear and UNI2-h ACMIL-MLP models (D). Figure partially created in BioRender [https://biorender.com/f125abi].

**Table 1:** Performance metrics of WSI deep learning models for endometrial live birth outcome prediction.

Performance Metric ± SD	ResNet-18 WSI	UNI2-h GAP-Linear WSI	UNI2-h GAP-MLP WSI	UNI2-h ACMIL-Linear WSI	UNI2-h ACMIL-MLP WSI
***Internal Testing Set***					
**Sensitivity**	0.727 ± 0.105	0.636 ± 0.315	0.727 ± 0.141	0.636 ± 0.200	0.636 ± 0.148
**Specificity**	0.800 ± 0.136	0.600 ± 0.235	0.700 ± 0.166	0.650 ± 0.197	0.750 ± 0.137
**PPV**	0.667 ± 0.165	0.467 ± 0.145	0.571 ± 0.073	0.500 ± 0.169	0.583 ± 0.160
**NPV**	0.842 ± 0.041	0.750 ± 0.106	0.824 ± 0.075	0.765 ± 0.086	0.789 ± 0.052
**F1**	0.696 ± 0.055	0.538 ± 0.184	0.640 ± 0.050	0.560 ± 0.073	0.608 ± 0.075
**Accuracy**	0.774 ± 0.063	0.613 ± 0.056	0.710 ± 0.071	0.645 ± 0.071	0.710 ± 0.068
**Balanced Accuracy**	0.764 ± 0.042	0.618 ± 0.057	0.714 ± 0.046	0.643 ± 0.045	0.693 ± 0.060
**AUC**	0.836 ± 0.025	0.627 ± 0.066	0.741 ± 0.030	0.727 ± 0.031	0.750 ± 0.066
***External Test Set***					
**Sensitivity**	1.000 ± 0.365	0.778 ± 0.292	0.556 ± 0.216	0.444 ± 0.333	0.556 ± 0.235
**Specificity**	0.083 ± 0.309	0.583 ± 0.377	1.000 ± 0.121	0.917 ± 0.382	1.000 ± 0.085
**PPV**	0.450 ± 0.177	0.583 ± 0.197	1.000 ± 0.168	0.800 ± 0.320	1.000 ± 0.316
**NPV**	1.000 ± 0.409	0.778 ± 0.352	0.750 ± 0.106	0.688 ± 0.252	0.750 ± 0.086
**F1**	0.621 ± 0.209	0.667 ± 0.167	0.714 ± 0.149	0.571 ± 0.198	0.714 ± 0.259
**Accuracy**	0.476 ± 0.090	0.667 ± 0.142	0.810 ± 0.094	0.714 ± 0.109	0.810 ± 0.105
**Balanced Accuracy**	0.542 ± 0.093	0.681 ± 0.119	0.778 ± 0.103	0.681 ± 0.083	0.778 ± 0.119
**AUC**	0.444 ± 0.140	0.815 ± 0.164	0.898 ± 0.051	0.778 ± 0.075	0.870 ± 0.095

PPV = positive predictive value; NPV = negative predictive value; AUC = area under the receiver operating characteristic curve

#### 1. ResNet-18 model with end-to-end training.

The ResNet-18 ensemble model trained on the internal natural cycle WSI dataset (ResNet-18 WSI model) demonstrated strong predictive capability when evaluated on the internal testing set (AUROC = 0.84 ± 0.03; balanced accuracy = 76.36 ± 4.23%) (Fig 2B). However, its performance on the external phase-based testing cohort collapsed rapidly during training, resulting in a final ensemble model achieving an AUROC of only 0.44 ± 0.14 and a balanced accuracy of 54.17 ± 9.31%, representing a poorer performance towards the external phase-based testing setting (Fig 2B; S2A Fig). DeLong test further indicated that the internal testing AUROC was significantly above that of a chance-level random classifier (*P* < 0.001), while the external phase-based testing AUROC remained not significantly different from chance level (*P* = 0.738).

#### 2. Frozen UNI2-h feature extractor with global average pooling.

The ensemble model utilizing the frozen UNI2-h feature extractor with a standard linear classifier head (UNI2-h GAP-Linear WSI model) indicated a limited overall predictive capability (Fig 2C). For the internal outcome-based testing cohort, the model achieved a non-significant AUROC of 0.63 ± 0.07 (*P* = 0.258) with a balanced accuracy of 61.82 ± 5.74%. A higher performance was observed in the external phase-based testing cohort, where the model has yielded a significant AUROC of 0.82 ± 0.16 (*P* = 0.002) with a balanced accuracy of 68.06 ± 11.86%.

Subsequently, the impact of coupling the feature extractor with a non-linear MLP classifier head (UNI2-h GAP-MLP WSI model) was evaluated (Fig 2C). While training trajectories across the internal cohorts remained generally consistent between both classifiers, the MLP variant demonstrated more rapid convergence on the external cohort during the training session (S2B Fig). For the internal testing cohort, the model achieved a significant AUROC of 0.74 ± 0.03 (*P* = 0.034) with a balanced accuracy of 71.36 ± 4.61%. Upon external phase-based testing, the model also yielded a significant AUROC of 0.90 ± 0.05 (*P* < 0.001) with a balanced accuracy of 77.78 ± 10.33%.

#### 3. Frozen UNI2-h feature extractor with weighted attention aggregation.

The application of the ACMIL aggregation strategy was further evaluated with either a standard linear classifier head or an MLP head (Fig 2D). On the internal testing cohort, the model coupled with a linear head (UNI2-h ACMIL-Linear WSI model) achieved a significant AUROC of 0.73 ± 0.03 (*P* = 0.034) with a balanced accuracy of 64.32 ± 4.52%. The model exhibited a comparable generalizability on the external phase-based test set, which yielded an AUROC of 0.78 ± 0.08 (*P* = 0.012) with a balanced accuracy of 68.06 ± 8.31%.

The model with ACMIL aggregation strategy utilizing the non-linear MLP classifier head (UNI2-h ACMIL-MLP WSI model) demonstrated significant and elevated AUROC values compared to random (Fig 2D). For the internal outcome-based testing cohort, this approach achieved an AUROC of 0.75 ± 0.07 (*P* = 0.031) with a balanced accuracy of 69.32 ± 6.03%. Upon external phase-based testing, the model maintained generally comparable to its GAP-based counter version, with an AUROC of 0.87 ± 0.09 (*P* < 0.001) and a balanced accuracy of 77.78 ± 11.87%. Model’s training trajectories did not indicate a clear advantage in convergence or stability for the ACMIL implementation compared with GAP (S2C Fig).

To further investigate the attention-weighting patterns of the ACMIL framework, we visualized the attention weights directly on the WSIs to identify whether particular histological regions were consistently prioritized by the model. Qualitative visual inspection revealed relatively sparse attention distribution without a strong and clear concentration towards a specific, recognizable morphological structure (S3 Fig).

### Performance of deep learning models trained on LE-containing regions

To address the concern that WSI-based approaches may dilute the existence of LE features, we developed biologically-targeted models trained exclusively on manually annotated LE regions (Fig 3A). Predictive performance was assessed across two distinct models trained with the LE-containing patches (namely ResNet-18 LE and UNI2-h LE models), with detailed performance metrics summarized in Table 2. Compared with the ResNet-18 WSI model, the ResNet-18 LE model has demonstrated a less divergent AUROC pattern across the internal outcome-based and external phase-based testing cohorts (Fig 3B; S2D Fig). For the internal testing set, the ResNet-18 LE model achieved a non-significant AUROC of 0.71 ± 0.03 (*P* = 0.090), with a balanced accuracy of 55.71 ± 5.75%. In the external phase-based testing cohort, the model achieved a significant AUROC of 0.80 ± 0.16 (*P* = 0.033) and a balanced accuracy of 70.00% ± 12.30%.

**Fig 3:**
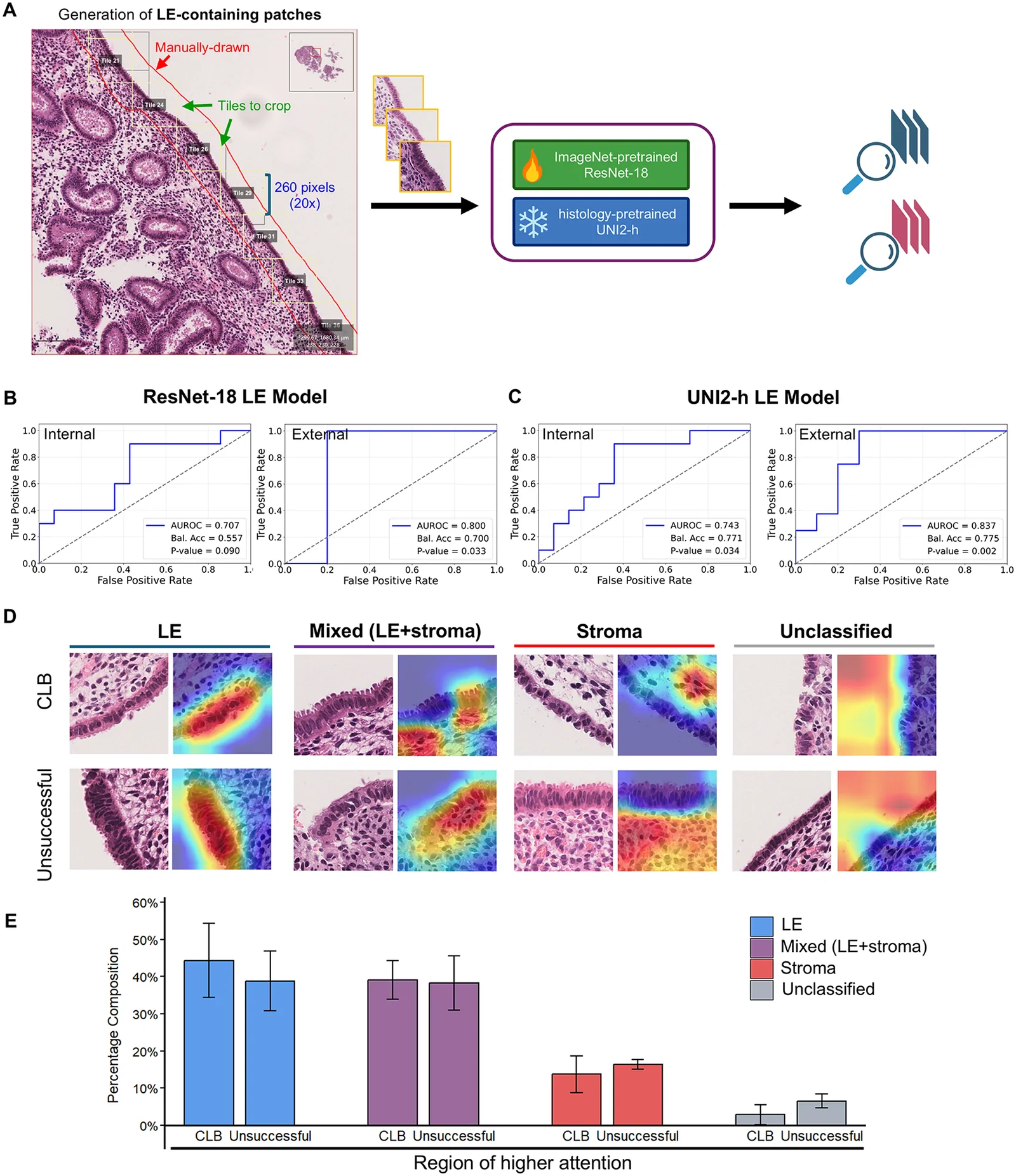
LE-targeted endometrial live birth outcome prediction models. (**A**) Schematic diagram showing the establishment of deep learning models trained on LE-containing regions. (**B**, **C**) ROC curves comparing model performance on the internal outcome-based (left) and external phase-based (right) testing cohorts for the LE-targeted ResNet-18 model (B) and LE-targeted UNI2-h model with an MLP classifier head (C). (**D**) Representative Grad-CAM visualizations from correctly predicted CLB and unsuccessful outcome cases. Higher attention areas are illustrated in warmer colours and lower attention areas are illustrated in cooler colours. (**E**) Semi-quantitative analysis of the average attention tendency of the models. Data are presented as the averaged percentage composition of attention categories from top-3 performing models within the 10-fold ensemble [(LE alone (blue), LE+stroma (purple), stroma alone (red), and unclassified (grey)], presented in Mean ± SD. Figure partially created in BioRender [https://biorender.com/f125abi].

**Table 2:** Performance metrics of LE-focused deep learning models for endometrial live birth outcome prediction.

Performance Metric ± SD	ResNet-18 LE	UNI2-h LE
***Internal Testing Set***		
**Sensitivity**	0.400 ± 0.181	0.900 ± 0.175
**Specificity**	0.714 ± 0.136	0.643 ± 0.158
**PPV**	0.500 ± 0.098	0.643 ± 0.090
**NPV**	0.625 ± 0.081	0.900 ± 0.094
**F1**	0.444 ± 0.086	0.750 ± 0.097
**Accuracy**	0.583 ± 0.052	0.750 ± 0.081
**Balanced Accuracy**	0.557 ± 0.057	0.771 ± 0.080
**AUC**	0.707 ± 0.027	0.743 ± 0.086
***External Test Set***		
**Sensitivity**	1.000 ± 0.159	0.750 ± 0.281
**Specificity**	0.400 ± 0.320	0.800 ± 0.151
**PPV**	0.571 ± 0.133	0.750 ± 0.076
**NPV**	1.000 ± 0.411	0.800 ± 0.147
**F1**	0.727 ± 0.083	0.750 ± 0.172
**Accuracy**	0.667 ± 0.142	0.778 ± 0.079
**Balanced Accuracy**	0.700 ± 0.123	0.775 ± 0.094
**AUC**	0.800 ± 0.164	0.837 ± 0.063

PPV = positive predictive value; NPV = negative predictive value; AUC = area under the receiver operating characteristic curve

The UNI2-h LE model also yielded performance comparable to its WSI variant (Fig 3C; S2D Fig). On the internal outcome-based testing cohort, the model achieved a significant AUROC of 0.74 ± 0.09 (*P* = 0.034) with a balanced accuracy of 77.14 ± 7.98%. Upon external phase-based testing, the model’s AUROC maintained at 0.84 ± 0.06 (*P* = 0.002) with a balanced accuracy of 77.50 ± 9.39%.

To further examine the spatial localization of model attention, Grad-CAM was utilized to visualize the high-attention regions driving the predictions of the three top-performing ResNet-18 LE models within the 10-fold ensemble, defined by their averaged AUROCs (0.79, 0.78, and 0.77). The resulting heatmaps were manually categorized according to the predominant localization of the highlighted compartments: (1) LE alone (attention primarily on LE), (2) mixed (attention spanning both LE and peri-LE stroma without clear preference), and (3) peri-LE stroma alone (attention primarily on peri-LE stroma), and (4) unclassified regions, including luminal spaces and potential artifacts (Fig 3D). Semi-quantitative analysis of the top-3 models showed that regions of higher attention were predominantly associated with LE-related compartments rather than peri-LE stroma-alone or unclassified regions. When stratified by predicted pregnancy outcome, patches showing LE alone and mixed attentions remained the two major categories in both CLB and unsuccessful cases, accounting for 44.33 ± 9.98% and 39.03 ± 5.20% of patches in CLB cases, and 38.83 ± 8.02% and 38.25 ± 7.34% in unsuccessful cases, respectively (Fig 3E). In contrast, patches showing high attention on peri-LE stroma alone were less frequent, accounting for 13.75 ± 4.92% and 16.37 ± 1.31% of patches in CLB and unsuccessful cases, respectively. Unclassified patches with attention restricted to empty luminal spaces lacking clear biological relevance, accounted for only a minor proportion in both groups (2.89 ± 2.68% and 6.55 ± 1.89% for CLB and unsuccessful cases, respectively). These results highlighted that Grad-CAM’s localization-based visualization was more focused on LE region.

### Comparisons of histology model performances

Paired DeLong tests were used for pairwise comparisons among the individual histology model configurations (S4 Fig). In the internal outcome-based testing cohort, none of the comparisons reached statistical significance after Benjamini-Hochberg adjustment. The two largest internal AUROC differences were between specific model configurations. ResNet-18 WSI and UNI2-h GAP-MLP WSI differed by ΔAUROC = 0.096, comparing end-to-end training with a foundation model-based pipeline. UNI2-h GAP-Linear WSI and UNI2-h GAP-MLP WSI differed by ΔAUROC = 0.114, comparing linear and MLP classifier heads. The remaining internal comparisons showed smaller differences, with the smallest difference observed between UNI2-h GAP-MLP WSI and UNI2-h ACMIL-MLP WSI (ΔAUROC = 0.009), comparing GAP and ACMIL aggregation. In the external phase-based testing cohort, UNI2-h GAP-MLP WSI achieved a significantly higher AUROC than ResNet-18 WSI, comparing the foundation model-based pipeline with end-to-end training (ΔAUROC = 0.454, *P* = 0.004). The remaining external comparisons were not statistically significant, with ΔAUROC ranging from 0.028 to 0.093. The smallest external difference was again observed between UNI2-h GAP-MLP WSI and UNI2-h ACMIL-MLP WSI (ΔAUROC = 0.028).

### Model calibration for probability prediction

Calibration curves and Brier scores were presented for each histology model separately in the internal outcome-based testing cohort and the external phase-based testing cohort (S5 Fig). The ResNet-18 WSI model showed the largest internal-external discrepancy, with the lowest Brier score in the internal testing cohort (0.161) but the highest Brier score in the external phase-based testing cohort (0.331), consistent with its divergent discriminative performance across the two testing settings. Among the UNI2-h WSI feature-based pipelines, Brier scores were generally within a narrower range. The UNI2-h ACMIL-MLP WSI model showed the lowest internal Brier score among the UNI2-h WSI models (0.205), while the UNI2-h GAP-MLP WSI model showed the lowest external Brier score (0.143). For the LE-focused models, the ResNet-18 LE model yielded Brier scores of 0.224 and 0.239 in the internal and external testing cohorts, respectively, while the UNI2-h LE model yielded Brier scores of 0.213 and 0.193, respectively.

### Multimodal integration of histology-based model outputs and clinical variables

To assess the potential benefit of multimodal integration, logistic regression models were constructed using predicted probabilities from the UNI2-h GAP-MLP WSI and UNI2-h LE pipelines on the internal outcome-based cohort, with or without maternal age (Age), BMI, endometrial thickness (EnTh), and E2 and P4 levels (detailed metrics in S4 Table). A model integrating the WSI and LE outputs achieved an AUROC of 0.80 (*P* = 0.006) with a balanced accuracy of 63.57% (Fig 4A). When Age, BMI, EnTh, E2, and P4 were further incorporated, the integrative seven-factor model achieved an AUROC of 0.77 (*P* = 0.034) with a balanced accuracy of 70.71% (Fig 4B). The clinical metadata-only model incorporating Age, BMI, EnTh, E2, and P4 yielded a non-significant AUROC of 0.52 (*P* = 0.903) with a balanced accuracy of 52.73% (Fig 4C). Two simpler Age + BMI + EnTh and E2 + P4 clinical metadata-only models were also evaluated, with non-significant AUROCs of 0.541 and 0.532, respectively (S6A, S6B Fig). In paired comparisons, both the WSI + LE model and the integrative seven-factor model achieved significantly improved discriminatory ability than the five-factor clinical metadata-only model (*P* = 0.026 for both comparisons). No significant difference was reached between the seven-factor and the WSI + LE models (*P* = 0.607).

**Fig 4:**
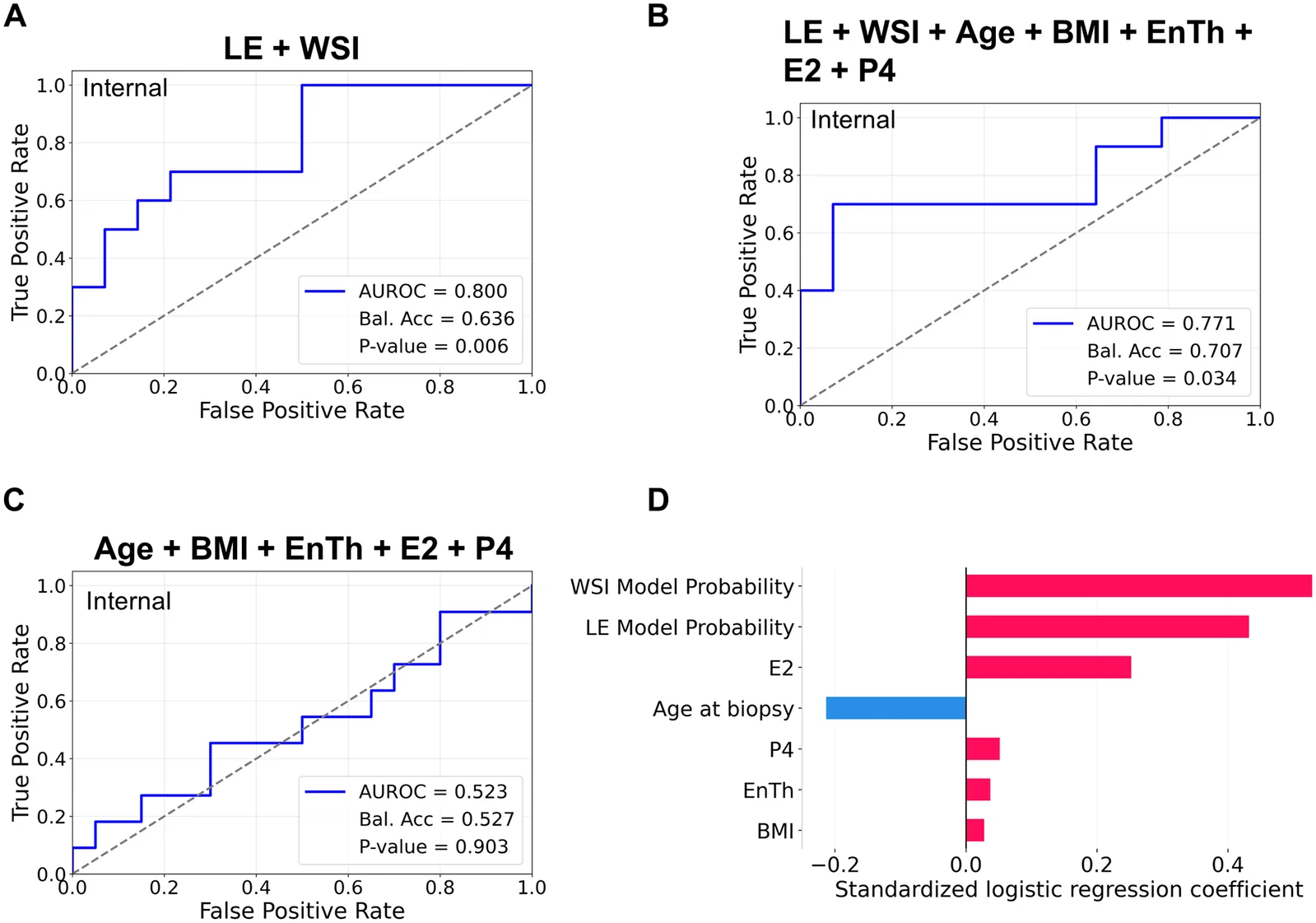
Multimodal integration of histology-based models with clinical and hormonal variables. ROC curves for the histology-only WSI + LE model (**A**), the integrative seven-factor LE + WSI + Age + BMI + EnTh + E2 + P4 model (**B**), and the Age + BMI + EnTh + E2 + P4 five-factor clinical metadata-only model (**C**), evaluated in the internal outcome-based testing cohort. (**D**) Standardized coefficients of the integrative seven-factor logistic regression model, showing the relative direction and magnitude of each feature’s contribution to the model output.

To further examine feature weighting patterns within the multimodal framework, standardized logistic regression coefficients and SHAP-based feature impacts were visualized. The standardized coefficients showed that the WSI model probability (0.529) and LE model probability (0.432) had the largest positive contributions to the model output, followed by E2 (0.253) and the maternal age with a negative weight (-0.213). Compared to the other features, P4 (0.052), EnTh (0.037), and BMI (0.028) contributed minimally with relatively lower impact (Fig 4D). Consistent with this pattern, SHAP analysis from either internal testing (S6C Fig) or training cohort (S6D Fig) demonstrated a broader distribution of feature impact for the WSI- and LE-based probabilities, smaller effects from E2 and maternal age, and minimal effects from P4, EnTh, and BMI. Together, these results summarized the relative contribution patterns of histology-based and clinical inputs within the multimodal framework.

## Discussion

This study explored the application of deep learning to predict cumulative live birth outcomes using human endometrial H&E WSIs. The reported ImageNet 1K-pretrained HRT model [27] was initially tested with our natural cycle endometrial WSIs. Even though patch preparation was matched to its published settings, the model showed poor predictive power. This limited transferability likely reflected the residual domain shift, biological differences between HRT and natural-cycle endometrium, and sample-size uncertainty, rather than implementation settings alone. The broader medical-imaging literature similarly emphasizes that model reliability is affected by pipeline factors, including sample preparation, patch generation, preprocessing, and validation designs [41,42]. Such domain shift might have been exacerbated by the small development cohort [27], further compromising stability and cross-institutional practicality. HRT samples form distinct sub-clusters from natural-cycle counterparts, with significant transcriptional shifts in the endometrial epithelial populations [43]. Histological studies similarly demonstrates HRT affects the thickness and the sizes of glandular and vascular space [29,44], further widening the discrepancy between the reported model and the test set.

In this study, two model development strategies were evaluated for cumulative live birth prediction using samples from natural cycles. The CNN-based ResNet-18 model was utilized as an end-to-end training pipeline due to its efficient and well-established architecture for medical image recognition tasks [45]. ResNet-18’s simplicity also made it practical for limited-data settings, where overfitting is a major concern [26,46]. In parallel, UNI2-h [21,47] was selected as one of the newest publicly accessible histopathology-pretrained foundation models at the time of our model development. Its ViT-Huge architecture and large-scale histopathology pretraining enable more complex histological inference. Nevertheless, its pretraining focused predominantly on cancer data, but not endometrial analysis or fertility outcomes. Thus, we evaluated whether frozen UNI2-h could be repurposed for non-malignant endometrial live-birth prediction.

The two approaches exhibited different performance tendencies. While the ResNet-18 model trained on internal natural cycle samples achieved relatively high predictive performance within the internal outcome-based testing cohort, its performance collapsed to near-random levels in the external phase-based testing cohort. This discrepancy may arise from two non-mutually exclusive factors. First, despite standardizing patches by pixel dimensions and physical coverage based on scanner-specific mpp metadata, residual upstream differences like tissue processing, sectioning, staining, scanner platforms may still have introduced institution-specific image characteristics [41,42]. Such effects may particularly impact end-to-end training, which can learn center-specific patterns from limited single-center data. Second, the external cohort lacked IVF outcome-based validation, instead evaluating receptive LH + 7 versus non-LH + 7 phase discrimination. The performance gap may reflect cross-institutional variation and limited target transferability, potentially similar to the previously assessed HRT model. Further outcome-matched validation with stronger statistical power is required.

In comparison, the UNI2-h feature-based pipeline completely lacks the ability to fine-tune its feature extractor’s logic. Despite, the pipeline eventually demonstrated models with less divergent performances across the internal outcome-based and external phase-based testing cohorts. With the standard linear-probe design, the UNI2-h GAP-Linear underperformed on the internal outcome-based test cohort, contrasting with the effective linear-probe performance reported in the original UNI benchmark [21]. Notably, healthy superficial endometrium and the subtle fertility-associated morphological features are likely underrepresented in UNI2-h’s knowledgebase of training. Li et al. [48] proposed that linear probes may be insufficient when the target differs substantially from the pretraining patterns. We therefore implemented a non-linear MLP head to better adapt UNI2-h-derived features to the endometrial fertility-oriented task. As expected, MLP-head models achieved higher AUROCs than their linear-head counterparts. Specifically, the UNI2-h GAP-MLP achieved significance in both internal and external cohorts, unlike the linear variant (external-only). This confirmed that frozen UNI2-h representations with MLP head are feasible for non-malignant endometrial live birth prediction without feature-extractor modification. Nevertheless, DeLong comparisons showed no significant difference between classifier heads, suggesting potential MLP utility without establishing statistical superiority given the current testing sample size. External generalizability for live birth prediction would require larger, outcome-matched cohorts. Beyond classifier-head adaptation, pretrained model selection may also influence downstream performance. Recent representational-similarity analysis showed that pathology foundation models (e.g., UNI2-h and Virchow2) extract divergent representations from the same histological images despite similar ViT-Huge architectures [49]. Whole-slide pretrained models, such as TITAN and Prov-GigaPath, further broaden the available strategies by incorporating broader slide-level context [50]. Thus, future comparisons across different feature-extraction strategies are essential to identify the optimal starting point for endometrial live birth-oriented analysis.

The study further implemented the ACMIL attention strategy [36] to evaluate whether prioritizing informative WSI regions prevents the dilution of critical features, a method known to improve performance in cancer-oriented studies [21,23,51]. However, comparable AUROC performance was achieved in GAP-MLP and ACMIL-MLP-based models, indicating ACMIL did not clearly outperform GAP. Calibration analysis provided a probability-level perspective of model performance beyond binary discrimination [52]. The UNI2-h ACMIL-MLP model maintained relatively low Brier scores across both testing cohorts, whereas the UNI2-h GAP-MLP model showed the highest internal Brier score. The differential weighting mechanism of ACMIL likely down-weighted the less-informative patches, making it potentially more reliable for probabilistic estimation than global pooling. Therefore, the ACMIL-MLP design can be preferable in clinical settings requiring patient-level probability estimates. For cumulative live birth prediction, larger-cohort comparisons should evaluate the most suitable aggregation approach. Post hoc recalibration may further be applied to improve the reliability of probability estimates used for individualized risk counselling regarding the likelihood of live birth [53].

ACMIL attention was sparse without clustered or consistent concentration within a specific histological compartment. This may be compatible with live birth prediction, where decision-relevant morphology may be more spatially distributed than in cancer-detection tasks [23]. Therefore, sparse weighting may reflect endometrial biopsy heterogeneity and not biologically uninformative. For example, asynchronous glands with mixed menstrual-stage epithelia have been observed in otherwise normal endometrial biopsies [54–56], which complicate phase assessment. Such within-slide heterogeneity may likewise affect attention interpretation in this fertility-oriented task. Further improvement requires deeper biological understanding of endometrial morphology and quantification of high-attention regions. One practical extension would be to quantify the interpretable histomics-centred features, such as nuclear morphology, cellular density, glandular architecture, and spatial cell-type relationships [57] in ACMIL-weighted patches, assessing whether high-attention regions are statistically associated with measurable morphological characteristics beyond visual inspection. Recent studies have demonstrated the use of AI-assisted endometrial cell annotation and targeted morphological analysis with explainable metrics such as epithelial-to-stromal ratio [58] and peri-gland stroma nuclear size [59]. However, no broadly available and cross-institutionally validated model yet reliably annotates LE, glandular epithelium, and stroma, while manual endometrial WSIs annotation remains impractical. Developing compartment-aware endometrial annotation models alongside outcome-oriented deep learning prediction models would enable objective attention-weighted histomics, interpretable handcrafted-feature baselines, and scalable ROI-based analysis. Complementing morphology and histomics-based approaches, recent work has aligned AI prediction heatmaps with Visium spatial transcriptomic profiles to validate molecular features associated with model-predicted regions [60]. Applying a similar strategy in endometrium could help determine whether attention patterns correspond to distinct cell states and potentially gene-expression profiles.

The LE-focused pipeline of analysis represents a manually defined, biologically informed ROI-based modelling strategy. The LE is the primary maternal-embryo interface during early implantation. Mouse *in vivo* studies, have highlighted that the LE undergoes morphological reshaping for implantation, affecting subcellular features including apical microvilli protrusions, apical tight junctions, and basolateral membranes [61–63]. However, capturing LE within a standard automated WSI patch generation pipeline is challenging because LE is sparse as compared to other cell types in endometrium. While only a minority of patients lacked identifiable LE, our dataset of LE-containing patches was substantially smaller than that of the standard WSI-based patches, with training set to be approximately 25 times smaller. The actual existence of LE within the WSI pipeline could be further compromised since the LE naturally has higher chance of falling into edge-case tiles dominated by empty spaces, which were discarded during the upstream patch generation procedures.

Beyond the LE, the adjacent peri-LE stroma may also represent a potentially underrepresented region worth specific emphasis. Histologically, the dense superficial stroma directly beneath the LE is the stratum compactum, whereas the deeper looser region is the stratum spongiosum [64]. Peri-LE stroma remained understudied until recent spatial transcriptomics suggests stromal WNT and NOTCH expressions may vary with distance from the luminal surface [65]. Therefore, our LE-containing ROI design was intended to enrich both LE and the potentially unique peri-LE stroma for separate evaluation. Surprisingly, despite the massive disparity in data volume of learning, models trained exclusively on LE-containing regions have achieved performance generally comparable to the WSI models. Together with the established biological roles of the LE and adjacent peri-LE stroma, these findings align with the expectation that this compartment represents a biologically enriched, morphologically informative reservoir for fertility-oriented endometrial modelling.

To visualize the morphological regions that were likely associated with model predictions, we employed Grad-CAM on the three best-performing LE-focused ResNet-18 models. This patch-level attention visualization was not extended to UNI2-h feature-based pipeline because reliable pixel-level saliency was difficult under slide-level feature aggregation. Semi-quantitative review showed that attention more often targeted LE-containing regions, either mainly the LE compartment or jointly with adjacent peri-LE stroma, while peri-LE stroma alone was less frequent. Unclassified artifacts or lumen-dominant regions were relatively rare. Thus, this localization tendency was consistent with the intended LE-focused design. Nevertheless, Grad-CAM provides only approximate spatial localization and does not specify the underlying mechanistic drivers. Reliable automated endometrial compartment annotation, together with more standardized attention quantification methods, would be essential for broader implementation of this interpretability framework and for more informative assessment of attention tendencies. Potentially, a multiscale design allowing more variable peri-LE context may also improve model robustness [57,66], as the physical boundary of the peri-LE stroma compartment has not yet been clearly defined by a fixed depth or anatomical cutoff [67,68].

Finally, multimodal integration was performed to explore the potential benefit of combining two histology-based models covering different regions of focus, with maternal clinical (age, BMI, and EnTh) and hormonal variables (E2 and P4). Such multimodal designs are increasingly explored in studies aiming to develop clinically useful predictive models [69]. Both WSI + LE and seven-factor models significantly outperformed chance and the clinical metadata-only model (Age + BMI + EnTh + E2 + P4). Standardized coefficients and SHAP analyses consistently ranked the two histology-model probabilities as the most impactful features. E2 and maternal age had small positive and negative associations, respectively; P4, EnTh, and BMI contributed minimally.

The inverse maternal age-live-birth association aligned with the known age-related decline [70]. The positive E2 and limited P4 associations were biologically plausible, consistent with a study that midluteal E2 levels were significantly associated with live birth rates once a minimum P4 level is reached [71]. The association between BMI and live birth remains cohort-dependent and controversial [72,73]. Limited P4 and EnTh weights do not imply biological irrelevance. Both variables are known to have minimal thresholds pregnancy supports [9,71,74]; thereby, a simpler threshold-based implementation may be more appropriate. Overall, these findings highlight the dominant contribution of histology-based predictions while demonstrating feasibility of incorporating complementary clinical variables. Future studies may explore earlier-stage multimodal integration to capture non-linear morphological–clinical relationships [57].

This study has several limitations. First, the patient-level sample size remained small although our cohort was larger than that of our previous HRT model [27]. Freezing the UNI2-h feature extractor reduced training complexity, but the low testing cohorts still weaken performance stability. Larger prospective cohorts are required to confirm the most suitable modelling strategy before wider implementation. Second, the external cohort was outcome-mismatched. Therefore, external performance should not be interpreted as direct validation of live birth prediction. Outcome-matched multicenter cohorts incorporating harmonization approaches (e.g., StainGAN) [75] are required to evaluate cross-center generalizability and mitigate technical variations. Third, the interpretability analyses remain limited to approximate spatial localization and could not support precise cellular or mechanistic interpretation from Grad-CAM saliency maps alone [76,77]. The manually defined LE workflow and lack of a validated endometrial compartment annotator further constrained scalable histomic analysis and the inclusion of a handcrafted morphological baseline for comparison.

From a clinical perspective, this predictive framework is best positioned as a supportive tool for pathologists and IVF clinicians when considering whether and when to proceed with embryo transfer. Unlike conventional dating, which is limited by biological and observer variability, this outcome-oriented pipeline offers a standardized, extendable, and quantifiable use of routine H&E images. On the other hand, the high cost of existing tests, such as transcriptomic array-based ERA, which costs US$800 or more per test, renders large prospective studies financially challenging [78]. The relatively lower cost of the proposed H&E image-based framework could practically facilitate larger validation and subsequent broader clinical implementation. Integrating biologically informed ROI modelling with automated histomic quantification could further enhance its comprehensiveness and interpretability. Of note, false positives could provide undue reassurance before embryo transfer, while false negatives could cause unnecessary delay or extra assessment. Thus, model thresholds should align with clinical priorities and undergo prospective validation. The clinical value of this framework relative to existing receptivity assessments also requires separate evaluation. With prospective validation and improved biological interpretation, it may enable more objective and personalized embryo-transfer planning.

In conclusion, this study showed that the histopathology pretrained UNI2-h foundation model could be effectively repurposed for endometrial fertility-related analysis without task-specific end-to-end training of the feature extractor. Although an endometrium-specific foundation model can be theoretically more ideal, such datasets are difficult to obtain in practice. Further outcome-matched external validation will be needed to formally assess generalizability for live-birth prediction. The LE-focused analyses further highlighted that a biologically informed design could retain performance comparable to WSI-based approaches despite using substantially fewer patches during development. Together, these findings support the LE and peri-LE regions and repurposed pathology models with ROI designs as promising starting points for endometrial histology analysis.

## Supporting information

S1 Fig HRT model performance on natural cycle samples. An ROC curve showing the performance of the reported 3-fold ensemble HRT-model on the natural cycle LH + 7 WSIs.
(TIF)

S2 Fig Summary of model predictive performance trajectories during training sessions. Summary figures illustrate progression of model predictive performance in AUROC (y-axis) over training epochs (x-axis). Performance is displayed for three cohorts including validation, internal testing, and external testing cohorts. All discussed models‘ performance trajectories were presented, including (**A**) ResNet-18 WSI model trajectory represented in blue solid line; (**B**) UNI2-h GAP-Linear and UNI2-h GAP-MLP model trajectories presented in blue and red solid lines; (**C**) UNI2-h ACMIL-Linear and UNI2-h ACMIL-MLP model trajectories presented in blue and red solid lines; (**D**) ResNet-18 LE and UNI2-h LE model trajectories presented in blue and red solid lines, with their WSI counter variants (ResNet-18 WSI and UNI2-h GAP-MLP model trajectories) plotted in purple and yellow dotted lines for easier comparison.
(TIF)

S3 Fig Visualization of attention distributions in the ACMIL-based UNI2-h model. Representative heatmaps display patch-level attention tendency on WSIs for a correctly predicted CLB case (**A**) and Unsuccessful (**B**) case. Warmer colors indicate higher attention weights.
(TIF)

S4 Fig Histology-based model pairwise performance comparison summary. Summary of model performance comparisons between specified models, specifically in the internal outcome-based (**A**) and the external phase-based (**B**) testing cohorts. Δ value presents difference of AUROC between model A and B within each specified pair. Formal statistics performed with DeLong test corrected for multiple comparison (p < 0.05 indicated with *).
(TIF)

S5 Fig Summary of per model calibration metrics. Calibration curves presented for each single reported models, including (**A**) ResNet-18 WSI, (**B**) UNI2-h GAP-Linear WSI, (**C**) UNI2-h GAP-MLP WSI, (**D**) UNI2-h ACMIL-Linear WSI, (**E**) UNI2-h ACMIL-MLP WSI, (**F**) ResNet-18 LE, and (**G**) UNI2-h LE model. Solid line presents model’s calibration curve on the internal test set; dotted line presents model’s calibration curve on the external set.
(TIF)

S6 Fig Additional multimodal model evaluations and feature-impact analysis. ROC curves for the Age + BMI + EnTh clinical metadata-only model (**A**) and the hormone-only E2 + P4 model **(B).** SHAP summary plot showing feature-contribution patterns across the internal test (**C**) and out-of-fold development samples (**D**) for the integrative seven-factor model. Positive SHAP values indicate a shift toward live birth prediction, negative SHAP values indicate a shift toward unsuccessful outcome prediction. Each dot represents one sample, with colour representing each samples’ original feature value, with red indicating a higher and blue representing a lower original value.
(TIF)

S1 Table Summary of clinical characteristics of the internal cohort by outcome group. (XLSX)

S2 Table Model training hyperparameter setting summary. (XLSX)

S3 Table Evaluation of the HRT-trained ResNet-18 model on the internal natural cycle cohort. (XLSX)

S4 Table Performance metrics of logistic regression models with multimodal integration. (XLSX)
