# Supplementary material for: Foundation model-powered deep learning of endometrial histology for predicting the cumulative live birth of an in vitro fertilization cycle
Source: PLOS Digit Health. 2026 Sep 28;5(9):e0001744. doi: 10.1371/journal.pdig.0001744 (PMC13618903; doi:10.1371/journal.pdig.0001744)
Supplement: Supplementary file 2 [file pdig.0001744.s012.docx]

**Response to Reviewers’ Comments**

**Reviewer #1:**

Comments to the Author

Good work.

Ans: We thank the reviewer for the positive comment of our revised manuscript and again appreciate the constructive comments provided in the previous round of review.

**Reviewer #2:**

Thank you for the substantial revisions. The manuscript has improved considerably, and the authors have addressed the major methodological and statistical concerns raised in the previous round. In particular, the addition of formal DeLong testing with multiple-comparison adjustment, calibration assessment, clearer discussion of limited sample size and model uncertainty, improved treatment of technical domain shift, and more cautious interpretation of model comparisons have strengthened the study.

One point would benefit from further clarification before publication.

The external cohort is now appropriately described as an “external phase-based testing” cohort rather than a true external validation cohort for cumulative live birth. However, the Abstract still presents the internal and external AUROC values in parallel, which may give readers the impression that the external cohort directly validates live-birth prediction. Because the external cohort distinguishes LH+7 from non-LH+7 samples rather than using actual IVF/live-birth outcomes, I suggest making this distinction explicit in the Abstract at the point where the external performance results are reported. The conclusion should also continue to avoid implying that these external results constitute direct clinical validation of live-birth prediction.

Aside from this point, the authors have responded comprehensively to the previous reviewer concerns, and the revised manuscript is substantially stronger.

Ans: We thank the reviewer for the positive comments, and we agree that the difference between the two testing cohorts should be made more explicit in the Abstract and Conclusion. The Abstract and Author Summary sections were revised to clarify the meanings of the two testing settings. The conclusion was also extended to additionally emphasize the need for outcome-matched external validation (lines 826-827), thereby avoiding misinterpretation of the current external validation.
