# Supplementary material for: Foundation model-powered deep learning of endometrial histology for predicting the cumulative live birth of an in vitro fertilization cycle
Source: PLOS Digit Health. 2026 Sep 28;5(9):e0001744. doi: 10.1371/journal.pdig.0001744 (PMC13618903; doi:10.1371/journal.pdig.0001744)
Supplement: Supplementary file 3 [file pdig.0001744.s011.docx]

**Response to Reviewers’ Comments**

**Reviewer: 1**

Comments to the Author

The paper presents an interesting application of deep learning and histopathology foundation models for predicting cumulative live birth outcomes from endometrial histology WSIs in IVF settings. The comparison between conventional ResNet-18 training and the UNI2-h foundation model is technically relevant, and the inclusion of luminal epithelium-focused analysis adds biological interpretability to the framework. The use of external validation, Grad-CAM analysis, multimodal integration, and discussion on domain transferability between cancer-oriented foundation models and non-malignant reproductive histology strengthens the manuscript’s contribution. The study also demonstrates good methodological transparency by reporting cross-validation strategies, preprocessing pipelines, and detailed evaluation metrics.

Ans: We thank the reviewer for the assessment of the study with positive comments on the application and design, and for identifying several areas to be further strengthened. The corresponding revisions are provided below.

Major comments:

1. The sample size remains relatively limited for developing robust histopathology AI models, especially for external validation and LE-focused experiments, which may affect statistical reliability and generalizability.

Ans: We agree with the reviewer about the sample size limitation and its potential effects on statistical confidence and model generalizability. The revised manuscript now more explicitly acknowledges the effects of the limited testing cohorts on performance stability and the need for larger prospective evaluation before wider implementation (lines 963-967). Formal DeLong tests with multiple-comparison adjustment were added for both chance-level and paired model comparisons, with the Results (line 478-527, 551-583, 610-625) and Discussion (line 736-748, 816-819, 830-834) revised to ensure appropriate statistical description and interpretations.

2. Although the authors discuss overfitting and domain shift, the manuscript would benefit from stronger analysis of stain normalization, scanner variability, and batch-effect mitigation strategies across institutions.

Ans: We agree that technical domain shift remains an important concern. We have made efforts in the standardization of patch images in resolution and physical tissue coverage levels to account for variations across scanning platforms and reducing avoidable variations (lines 305-342). In this revised version, we further recognized and addressed the lack of more comprehensive technical harmonization analysis and implementations in the Discussion. Manuscript now explicitly acknowledges that residual technical differences may still have introduced institution-specific characteristics to affect model evaluations. The suggested article was also incorporated (lines 704-716, 739-748). Advanced harmonization approaches were further discussed for future evaluation (lines 967-974).

3. The biological interpretation of the learned representations remains somewhat limited, as Grad-CAM only provides coarse localization rather than mechanistic insight into fertility-associated morphological phenotypes.

Ans: We agree and have substantially refined the interpretability claims. The Discussion was revised to emphasize that Grad-CAM provided only approximate spatial localization and did not specify the underlying mechanistic drivers (lines 928-942, 974-979). More objective next steps beyond visual inspection, including attention-weighted histomics-based approaches, were also discussed (line 852-868).

4. The multimodal analysis is also relatively simple, relying only on logistic regression with age and endometrial thickness, whereas additional reproductive and hormonal variables could further improve prediction.

Ans: We thank the reviewer for this suggestion. The multimodal analysis was expanded to include biopsy-day serum estradiol and progesterone levels, and BMI, with five logistic regression settings including a histology-only, an integrative histology-clinical, and three clinical metadata-only models (new Fig 4, S6 Fig, S4 Table, line 371-381). The model’s statistical analysis, updated feature weights were presented in the revised Results (line 641-699), and extended Discussions (lines 943-962) about the multimodal analysis’s interpretations in the context of live birth prediction were added in the revised manuscript.

5. The literature review should also be expanded to better position the work within computational pathology and histomics-based representation learning. The authors are encouraged to discuss and cite “Phenotyping of Histology Imaging Data with Histomics”.

Ans: We thank the reviewer for suggesting this highly relevant technical reference. The article was incorporated into the revised Discussion to better position the framework within histomics-based computational pathology, with expanded discussion on the benefits, feasibility, and potential applications of an objective histomics-based framework, within the context of fertility-oriented endometrial analysis (lines 852-868).

**Reviewer: 2**

Comments to the Author

This manuscript presents an interesting and clinically relevant application of deep learning and foundation models for predicting IVF cumulative live birth outcomes using endometrial histology whole-slide images. The comparison between conventional CNN-based approaches and the UNI2-h foundation model is valuable, and the biologically informed luminal epithelium (LE)-focused analysis adds originality to the study. The inclusion of external validation and explainability analyses are important strengths.

The manuscript is generally well written and technically sound overall. However, several methodological and interpretability concerns should be addressed before publication.

Overall, this is a promising and innovative study with meaningful translational potential. The concerns above appear addressable and would substantially strengthen the manuscript.

Ans: We thank the reviewer for the positive feedbacks. Refinements relevant to the raised methodological and interpretability concerns were explained as follows in point-by-point manner.

Major comments:

1. The external cohort labeling strategy requires further clarification and discussion. The external dataset did not use actual IVF/live birth outcomes but instead inferred labels based on LH timing. While biologically reasonable, this is not equivalent to true clinical live birth outcomes and may affect external validity and model performance interpretation.

Ans: We agree that the external testing cohort is biologically relevant but non-equivalent to internal cohort’s actual live birth outcomes. The Methods was refined to further clarify the difference between the two cohorts (lines 219-233). The manuscript was revised throughout to describe this cohort as “external phase-based testing”. The Discussion was extended (lines 744-748, 818-819, 967-974) to further acknowledge this target mismatch as one of the potential contributors to the reported performance discrepancies, and to emphasize the need for outcome-matched validations.

2. The relatively limited patient-level sample size raises concerns regarding overfitting and generalizability, particularly given the large number of extracted patches and multiple model configurations evaluated. Additional discussion regarding statistical uncertainty and robustness across institutions would strengthen the manuscript.

Ans: As addressed in our response to Reviewer 1 comment 1, we agree with the reviewer about the sample size limitation and have made revisions accordingly. The manuscript was revised to better acknowledge the potential effects, with emphasized need for larger prospective evaluations (lines 963-967). The limitation was further reflected in the additionally implemented formal statistical tests, with the Methods (lines 413-414, 426-431), Results (lines 478-527, 551-583, 610-625) and Discussion (lines 816-819, 830-834) revised to ensure appropriate statistical interpretations.

3. The manuscript reports AUROC and balanced accuracy values across models, but no formal statistical comparison between model performances is presented. Statistical testing comparing AUROCs or confidence interval overlap analysis would strengthen claims regarding superiority or robustness of specific architectures.

Ans: We thank the reviewer for recommending formal statistical comparisons. Statistical comparisons of model discriminative capabilities were performed using DeLong tests, covering two major settings to enable comparisons to chance-level random classifier and paired model-to-model comparisons (line 413-414, 426-431). The Results were also substantially revised throughout to ensure statistically appropriate descriptions (lines 478-527, 551-583, 610-625, 647-663). The Discussion was revised to avoid overstating statistical superiority based on numerical differences (lines 816-819, 830-834).

4. Although the Grad-CAM analysis is informative, the semi-quantitative categorization appears partially subjective. The manuscript would benefit from additional clarification regarding annotation criteria, reviewer blinding, and reproducibility of the interpretability assessment.

Ans: We agree with the reviewer’s concerns regarding the subjectivity and limited clarification of the Grad-CAM analysis. The Methods section was revised to provide clearer description of the observer blinding details, and the rationale for the four prespecified localization categories (line 348-363). As addressed in our response to Reviewer 1 Comment 3 and Reviewer 3 Comment 6, the Grad-CAM-related Results (lines 585-608) and Discussion (lines 935-937, 974-979) were refined to limit interpretation to approximate spatial localization and explicitly acknowledge Grad-CAM’s limitations.

5. The clinical applicability of this approach should be discussed in greater depth. It would be valuable to better contextualize how this workflow could realistically integrate into IVF practice and how it compares with currently available receptivity assessment tools.

Ans: We agree that the potential clinical application of the proposed framework required clearer discussion. The Discussion was expanded to position the framework as a supportive tool for pathologists and IVF clinicians, with further discussions of its potential benefits as compared to conventional dating and transcriptomic receptivity testing (ERA). The implications of prediction errors, threshold selection, and the need for prospective validations were also addressed (lines 980-999).

Minor comments:

1. Terminology regarding “live birth prediction,” “fertility prediction,” and “receptivity prediction” should be standardized throughout the manuscript for clarity.

Ans: The manuscript was revised to standardize terminologies, with the model’s task consistently described as live birth prediction throughout. Receptivity-related terminology was retained only when referring to the general biological concept. External testing cohort was corrected with description of phase-based prediction to better emphasize its inherent difference.

2. Additional discussion comparing UNI2-h with other recent pathology foundation models would help contextualize model selection.

Ans: We thank the reviewer for this suggestion. The revised Discussion further clarified that UNI2-h was selected as one of the newest publicly accessible histopathology-pretrained foundation models at the time of our model development (lines 725-735). Additional discussions were made, emphasizing the value and necessity of future further backbone comparisons (line 820-827).

3. Minor language editing and sentence shortening in several sections would improve readability.

Ans: We revised the manuscript throughout to shorten sentences, improved readability, and corrected for minor grammatical corrections.

**Reviewer: 3**

This manuscript presents an interesting and clinically relevant application of computational pathology by leveraging foundation models and deep learning for predicting cumulative live birth outcomes from endometrial histology. The comparison between conventional CNN-based approaches and the histopathology foundation model (UNI2-h), together with the biologically motivated luminal epithelium (LE)-focused analysis, provides novelty and translational potential. The inclusion of external validation and explainability analyses using Grad-CAM further strengthens the study. However, several methodological, statistical, biological, and presentation-related limitations need to be addressed before publication.
The manuscript would benefit from deeper methodological rigor, stronger biological interpretation, clearer discussion of limitations, and broader integration with existing quantitative pathology literature. In particular, the study positions itself within computational pathology but overlooks important literature on histomics and quantitative image phenotyping, which are highly relevant to the interpretation and biological validation of histology-based AI models.
Suggested references that should be discussed: "Phenotyping of Histology Imaging Data with Histomics",
"Radiomics in Medical Imaging: Methods, Applications, and Challenges"
These works provide important perspectives on quantitative tissue phenotyping, interpretable feature extraction, reproducibility, feature stability, and biological representation learning, all of which are directly relevant to the proposed endometrial histology analysis framework.

Ans: We thank the reviewer for the positive assessment comments and for identifying several important areas for improvement. The methodological, statistical, biological, interpretability, and limitation-related concerns are addressed individually in the responses below and corresponding revisions have been made throughout the manuscript. We also highly appreciate the two recommended references, which provided valuable perspectives on interpretable histomic quantification, and technical reproducibility. Both articles have been incorporated into the revised Discussion, to their most relevant sections (lines 712-716, 740-743, 852-856, 940-942, 960-962).

1. Limited Dataset Size Relative to Model Complexity
A major concern is the relatively small patient cohort used to train and evaluate extremely large deep learning models.
The training cohort consists of only 152 patients, while UNI2-h contains hundreds of millions of parameters. Although the feature extractor is frozen, the study still attempts to learn fertility-related representations from a limited number of cases.
The manuscript repeatedly attributes superior performance to foundation-model knowledge transfer, but it remains unclear whether observed improvements are statistically significant or merely reflect sampling variability.
The authors should discuss:
Dataset-size limitations more explicitly.
Risks of model instability and optimistic performance estimates.
Statistical power limitations.
Whether performance differences between models are statistically significant.
Confidence intervals alone are insufficient to demonstrate superiority.

Ans: As addressed in our response to Reviewer 1 Comment 1 and Reviewer 2 Comment 2 and 3, we agree that the dataset size is limited relative to model complexity. The Methods explicitly acknowledges both the large model size and the limited sample size. Thereby, all UNI2-h weights were kept completely frozen, as the reviewer have raised (lines 279-282). The manuscript was revised to better acknowledge the potential effects of the smaller sample size, with emphasized need for larger prospective evaluations (lines 963-967). The limitation was further reflected in the additional formal statistical tests and their interpretations in Results (line 478-527, 551-583, 610-625) and Discussion (lines 816-819, 832-834).

2. External Validation Design is Problematic
The external cohort does not contain actual IVF outcome labels.
Instead, fertility status is inferred from menstrual phase timing:
LH+7 → labeled as successful
LH+3, LH+5, LH+9, LH+11 → labeled as unsuccessful
This introduces a substantial conceptual issue.
The internal model is trained to predict cumulative live birth outcomes, whereas external validation evaluates the ability to distinguish endometrial phases.
These are fundamentally different prediction targets.
Consequently, the reported external AUROC values may not truly represent generalization for live birth prediction.
The manuscript should explicitly acknowledge this limitation and avoid presenting external performance as a direct validation of IVF outcome prediction.

Ans: As addressed in our response to Reviewer 2 Comment 1, we fully agree that the external phase-based testing cohort is biologically relevant but non-equivalent to internal cohort’s actual live birth outcomes. The Methods section was refined to clarify the differences in two cohort’s label definitions (lines 219-233). The manuscript was revised throughout to describe this cohort as external phase-based testing, with Discussions (lines 744-748, 815-819, 967-974) also extended to further acknowledge the effects of this target mismatch in relevance to model performance discrepancy, and to emphasize the need for outcome-matched validations).

3. Insufficient Biological Interpretation
Although Grad-CAM visualizations are provided, biological interpretation remains superficial.
The study concludes that LE-associated regions contribute to prediction, but does not explain:
Which morphological characteristics drive predictions.
Whether attention corresponds to known implantation biology.
Whether glandular, stromal, vascular, inflammatory, or epithelial features are implicated.
The discussion remains largely model-centric rather than biology-centric.
More detailed pathological interpretation is necessary to support clinical relevance.

Ans: We agree that the biological interpretation of the model predictions required further clarification. The Discussion was refined to better explain the implantation-related rationale for focusing on the LE and peri-LE stroma for fertility outcome prediction, particularly through extended discussion regarding the known histological and spatial-transcriptomic characteristics in relevance to the LE and peri-LE compartments (lines 869-890, 922-927). The Discussion also refined the description and interpretation of ACMIL attention patterns in relation to within-biopsy heterogeneity, with attention-weighted histomics explained as one practical extension for improving interpretability (line 844-868). As addressed in our response to Reviewer 1 Comment 3 and Reviewer 2 Comment 4, the manuscript was further revised in Discussion (lines 936-942, 974-979) to limit Grad-CAM’s interpretation to approximate spatial localization and avoid overstating mechanistic drivers.

4. Missing Quantitative Histomics Perspective
The manuscript treats the problem entirely as a deep-learning classification task.
However, computational pathology increasingly emphasizes interpretable quantitative tissue phenotyping through histomics-based approaches.
The authors should discuss how their deep learning framework relates to established histomic descriptors such as:
Nuclear morphology
Cellular density
Glandular architecture
Spatial organization
Tissue texture patterns
The manuscript would be significantly strengthened by discussing findings in the context of:
Phenotyping of Histology Imaging Data with Histomics
This reference provides an important framework for linking computational image features with biologically meaningful tissue phenotypes.

Ans: We appreciate the suggested reference and agreed such histomics perspective is highly relevant to improving the interpretability of the current framework. As also addressed by Reviewer 1 Comment 5, the histomics-based framework and the suggested article were incorporated into the revised Discussion. A practical attention-weighted histomics extension was proposed, and the need for compartment-aware endometrial annotation models was further highlighted to enable objective and scalable histomic analysis in practice (lines 852-868).

5. Lack of Comparison with Handcrafted Feature Approaches
The study compares multiple deep learning architectures but does not include any traditional quantitative pathology baseline.
A comparison against:
Histomics features,
Morphological descriptors,
Classical machine learning models,
would provide valuable insight into whether foundation models truly outperform interpretable quantitative approaches.
Without such comparison, it is difficult to determine the added value of the proposed deep learning framework.

Ans: We agree that a handcrafted morphological baseline would provide a valuable and interpretable comparison. Ideally, a predictive model based on low-dimensional metrics derived separately from the major endometrial compartments would provide a more appropriate morphological baseline for assessing the added value of the implemented deep learning model frameworks. However, in practice, reliable compartment-level endometrial annotation is not yet broadly available, while manual WSI annotation remains impractical at scale. We recognize that simpler morphological histomic features could potentially be extracted using existing H&E-pretrained models, if ignoring the biologically essential need of compartment awareness. However, to our knowledge, no universally established pipeline convention or evaluation framework currently exists for determining the reliability of such a morphological feature extraction workflow. Establishing a reliable endometrial histomics pipeline would therefore require substantial additional evaluation, particularly for using it as a standard baseline.

The revised Discussion therefore highlights the development of compartment-aware annotation models as a key step toward interpretable handcrafted-feature baselines and scalable ROI-based analysis (lines 852-864). The lack of a handcrafted morphological baseline is also explicitly acknowledged as a limitation (lines 976-979).

6. Interpretability Claims Are Overstated
The manuscript repeatedly emphasizes biological interpretability.
However, Grad-CAM alone does not provide mechanistic interpretability.
Grad-CAM merely highlights image regions associated with model decisions and does not explain:
Which features were learned,
Why predictions were made,
Whether highlighted regions are causally relevant.
The authors should moderate interpretability claims and acknowledge limitations of attention-based explanations.

Ans: As addressed in our response to Reviewer 1 Comment 3 and Reviewer 2 Comment 4, we agree that Grad-CAM does not provide mechanistic interpretability. The Grad-CAM-related Results and Discussion were revised to limit the interpretation to approximate spatial localization and to avoid inferring exact morphological features, cellular drivers, or causal mechanisms from the saliency maps alone (lines 935-940, 974-979). More objective attention quantification and attention-weighted histomics analysis were further discussed as practical extensions for more informative interpretation (lines 852-868, 936-942).

7. No Assessment of Model Calibration
Clinical deployment requires more than discrimination metrics.
The study reports:
AUROC
Accuracy
Balanced accuracy
but does not evaluate calibration.
Important metrics such as:
Calibration curves
Brier score
Expected calibration error (ECE)
should be considered.
A model with good AUROC may still generate poorly calibrated probabilities unsuitable for clinical decision-making.

Ans: We agree that calibration is important for evaluating probability-level predictions. Calibration curves and Brier scores were added for each histology model (new S5 Fig), assessed separately in the internal outcome-based and external phase-based testing cohorts (lines 431-434, 627-639). The Discussion was also extended to interpret the calibration findings, including the potentially greater probability reliability of ACMIL-MLP than GAP-MLP, and to discuss post hoc recalibration (lines 830-843). Expected calibration error was not additionally implemented because its bin-dependent estimation may be relatively unstable in the current small testing cohorts.

8. Limited Discussion of Reproducibility and Domain Shift
The study demonstrates substantial performance degradation of the HRT-trained model when applied to natural-cycle data.
This observation highlights a broader issue of domain shift.
However, the discussion does not sufficiently address:
Scanner variability
Staining variability
Institution-specific biases
Preprocessing variability
Batch effects
These challenges are central topics in quantitative imaging research.
The authors may benefit from discussing reproducibility principles described in:
Radiomics in Medical Imaging: Methods, Applications, and Challenges
which provides a comprehensive discussion of feature robustness, reproducibility, and external validation.

Ans: As addressed in our response to Reviewer 1 Comment 2, we agree that technical reproducibility and domain shift required clearer discussion. The study has made efforts to standardize patch resolution and physical coverage across scanning platforms (lines 305-342). Nevertheless, the revised Discussion acknowledges that residual differences may still have introduced institution-specific image characteristics (lines 740-748). The suggested quantitative-imaging reference was incorporated to support this discussion (lines 712-716). More advanced harmonization approaches were highlighted for future evaluation (lines 972-974).

9. Statistical Analysis Needs Strengthening
The manuscript reports mean ± SD values across folds.
However, no statistical comparisons are presented between:
ResNet-18 vs UNI2-h
GAP vs ACMIL
Linear vs MLP
WSI vs LE models
Without statistical testing, claims regarding superiority remain largely descriptive.
Appropriate significance testing or confidence interval comparisons should be considered.

Ans: As addressed in our response to Reviewer 2 Comment 3, we thank the reviewer for recommending formal statistical comparisons. Statistical comparisons of model discriminative capabilities were performed using DeLong tests, covering two major settings to enable comparisons to chance-level random classifier and paired model-to-model comparisons (line 426-431). The Results were also substantially revised throughout to report model-specific statistical significance, with several models showing discrimination significantly above chance and UNI2-h GAP-MLP WSI achieving a significantly higher external AUROC than ResNet-18 WSI (lines 478-527, 551-583, 610-625). Nevertheless, with most paired comparisons remaining non-significant, the Discussion was revised to avoid overstating statistical superiority based on numerical differences (lines 816-819, 830-834).

10. Clinical Utility Remains Unclear
Even the best-performing models achieve AUROCs around 0.84–0.90.
The manuscript does not explain:
How predictions would alter IVF decision-making.
Whether performance exceeds existing clinical assessment approaches.
Whether cost-benefit advantages exist.
How false-positive and false-negative predictions would affect patients.
The clinical translation pathway remains insufficiently developed.

Ans: As addressed in our response to Reviewer 2 Comment 5, we agree that the clinical translation pathway required clearer discussion. The revised Discussion positions the framework as a supportive tool when considering whether and when to proceed with embryo transfer, and discussed its potential value compared with conventional dating and endometrial receptivity analysis (ERA) test (lines 980-989). It also addresses the implications of false-positive and false-negative predictions, threshold selection, and the need for prospective validation and separate evaluation against existing receptivity assessments (lines 990-993).
