# Supplementary material for: Foundation model-powered deep learning of endometrial histology for predicting the cumulative live birth of an in vitro fertilization cycle
Source: PLOS Digit Health. 2026 Sep 28;5(9):e0001744. doi: 10.1371/journal.pdig.0001744 (PMC13618903; doi:10.1371/journal.pdig.0001744)
Supplement: Peer review history

# Peer review: Decision Letter 0

**DOI:** 10.1371/journal.pdig.0001744.r001

**Authors:** Feng Liu, Fnu Neha

14 Jun 2026

Response to Reviewers Revised Manuscript with Track Changes Manuscript **Journal Requirements:**

1. Please amend your detailed Financial Disclosure statement. This is published with the article. It must therefore be completed in full sentences and contain the exact wording you wish to be published.

a. State the initials, alongside each funding source, of each author to receive each grant.

b. State what role the funders took in the study. If the funders had no role in your study, please state: “The funders had no role in study design, data collection and analysis, decision to publish, or preparation of the manuscript.”

c. If any authors received a salary from any of your funders, please state which authors and which funders.

2.  We note that you have indicated that there are restrictions to data sharing for this study. For studies involving human research participant data or other sensitive data, we encourage authors to share de-identified or anonymized data. However, when data cannot be publicly shared for ethical reasons, we allow authors to make their data sets available upon request. For information on unacceptable data access restrictions, please see http://journals.plos.org/plosone/s/data-availability#loc-unacceptable-data-access-restrictions.

Before we proceed with your manuscript, please address the following prompts:

a) If there are ethical or legal restrictions on sharing a de-identified data set, please explain them in detail (e.g., data contain potentially identifying or sensitive patient information, data are owned by a third-party organization, etc.) and who has imposed them (e.g., a Research Ethics Committee or Institutional Review Board, etc.). Please also provide contact information for a data access committee, ethics committee, or other institutional body to which data requests may be sent.

b) If there are no restrictions, please upload the minimal anonymized data set necessary to replicate your study findings to a stable, public repository and provide us with the relevant URLs, DOIs, or accession numbers. Please see http://www.bmj.com/content/340/bmj.c181.long for guidelines on how to de-identify and prepare clinical data for publication. For a list of recommended repositories, please see https://journals.plos.org/plosone/s/recommended-repositories. You also have the option of uploading the data as Supporting Information files, but we would recommend depositing data directly to a data repository if possible.

Please update your Data Availability statement in the submission form accordingly.

3. Some material included in your submission may be copyrighted. According to PLOS’s copyright policy, authors who use figures or other material (e.g., graphics, clipart, maps) from another author or copyright holder must demonstrate or obtain permission to publish this material under the Creative Commons Attribution 4.0 International (CC BY 4.0) License used by PLOS journals. Please closely review the details of PLOS’s copyright requirements here: PLOS Licenses and Copyright. If you need to request permissions from a copyright holder, you may use PLOS's Copyright Content Permission form.

Please respond directly to this email or email the journal office and provide any known details concerning your material's license terms and permissions required for reuse, even if you have not yet obtained copyright permissions or are unsure of your material's copyright compatibility.

Potential Copyright Issues:

Fig 1,2,3: Please confirm whether you drew the images / clip-art within the figure panels by hand. If you did not draw the images, please provide (a) a link to the source of the images or icons and their license / terms of use; or (b) written permission from the copyright holder to publish the images or icons under our CC-BY 4.0 license. Alternatively, you may replace the images with open source alternatives. See these open source resources you may use to replace images / clip-art:

- https://commons.wikimedia.org

- https://openclipart.org/

**Additional Editor Comments (if provided)**

Comments from the Editorial Office: To ensure transparency, we are informing you that the previous Academic Editor acted as Reviewer 3 on your manuscript.

**Reviewers' Comments:**

**Comments to the Author**

1. Does this manuscript meet PLOS Digital Health’s publication criteria?

Reviewer #1: Yes

Reviewer #2: Partly

Reviewer #3: Yes

**********

2. Has the statistical analysis been performed appropriately and rigorously?

Reviewer #1: N/A

Reviewer #2: Yes

Reviewer #3: N/A

**********

3. Have the authors made all data underlying the findings in their manuscript fully available (please refer to the Data Availability Statement at the start of the manuscript PDF file)?

The PLOS Data policy

Reviewer #1: Yes

Reviewer #2: Yes

Reviewer #3: Yes

**********

4. Is the manuscript presented in an intelligible fashion and written in standard English?

Reviewer #1: Yes

Reviewer #2: Yes

Reviewer #3: Yes

**********

Reviewer #1: The paper presents an interesting application of deep learning and histopathology foundation models for predicting cumulative live birth outcomes from endometrial histology WSIs in IVF settings. The comparison between conventional ResNet-18 training and the UNI2-h foundation model is technically relevant, and the inclusion of luminal epithelium-focused analysis adds biological interpretability to the framework. The use of external validation, Grad-CAM analysis, multimodal integration, and discussion on domain transferability between cancer-oriented foundation models and non-malignant reproductive histology strengthens the manuscript’s contribution. The study also demonstrates good methodological transparency by reporting cross-validation strategies, preprocessing pipelines, and detailed evaluation metrics.

However, several aspects require further clarification and improvement before publication. The sample size remains relatively limited for developing robust histopathology AI models, especially for external validation and LE-focused experiments, which may affect statistical reliability and generalizability. Although the authors discuss overfitting and domain shift, the manuscript would benefit from stronger analysis of stain normalization, scanner variability, and batch-effect mitigation strategies across institutions. In addition, the biological interpretation of the learned representations remains somewhat limited, as Grad-CAM only provides coarse localization rather than mechanistic insight into fertility-associated morphological phenotypes. The multimodal analysis is also relatively simple, relying only on logistic regression with age and endometrial thickness, whereas additional reproductive and hormonal variables could further improve prediction.

The literature review should also be expanded to better position the work within computational pathology and histomics-based representation learning. The authors are encouraged to discuss and cite "Phenotyping of Histology Imaging Data with Histomics", which provides important insights into phenotype-driven histology representation learning, quantitative tissue characterization, and interpretable histopathological feature extraction. This reference would strengthen the manuscript’s discussion regarding biologically meaningful tissue phenotyping, representation-level learning, and morphology-driven computational pathology pipelines, particularly in the context of fertility-oriented endometrial analysis.

Reviewer #2: This manuscript presents an interesting and clinically relevant application of deep learning and foundation models for predicting IVF cumulative live birth outcomes using endometrial histology whole-slide images. The comparison between conventional CNN-based approaches and the UNI2-h foundation model is valuable, and the biologically informed luminal epithelium (LE)-focused analysis adds originality to the study. The inclusion of external validation and explainability analyses are important strengths.

The manuscript is generally well written and technically sound overall. However, several methodological and interpretability concerns should be addressed before publication.

Major comments:

The external cohort labeling strategy requires further clarification and discussion. The external dataset did not use actual IVF/live birth outcomes but instead inferred labels based on LH timing. While biologically reasonable, this is not equivalent to true clinical live birth outcomes and may affect external validity and model performance interpretation.

The relatively limited patient-level sample size raises concerns regarding overfitting and generalizability, particularly given the large number of extracted patches and multiple model configurations evaluated. Additional discussion regarding statistical uncertainty and robustness across institutions would strengthen the manuscript.

The manuscript reports AUROC and balanced accuracy values across models, but no formal statistical comparison between model performances is presented. Statistical testing comparing AUROCs or confidence interval overlap analysis would strengthen claims regarding superiority or robustness of specific architectures.

Although the Grad-CAM analysis is informative, the semi-quantitative categorization appears partially subjective. The manuscript would benefit from additional clarification regarding annotation criteria, reviewer blinding, and reproducibility of the interpretability assessment.

The clinical applicability of this approach should be discussed in greater depth. It would be valuable to better contextualize how this workflow could realistically integrate into IVF practice and how it compares with currently available receptivity assessment tools.

Minor comments:

Terminology regarding “live birth prediction,” “fertility prediction,” and “receptivity prediction” should be standardized throughout the manuscript for clarity.

Additional discussion comparing UNI2-h with other recent pathology foundation models would help contextualize model selection.

Minor language editing and sentence shortening in several sections would improve readability.

Overall, this is a promising and innovative study with meaningful translational potential. The concerns above appear addressable and would substantially strengthen the manuscript.

Reviewer #3: This manuscript presents an interesting and clinically relevant application of computational pathology by leveraging foundation models and deep learning for predicting cumulative live birth outcomes from endometrial histology. The comparison between conventional CNN-based approaches and the histopathology foundation model (UNI2-h), together with the biologically motivated luminal epithelium (LE)-focused analysis, provides novelty and translational potential. The inclusion of external validation and explainability analyses using Grad-CAM further strengthens the study. However, several methodological, statistical, biological, and presentation-related limitations need to be addressed before publication.

The manuscript would benefit from deeper methodological rigor, stronger biological interpretation, clearer discussion of limitations, and broader integration with existing quantitative pathology literature. In particular, the study positions itself within computational pathology but overlooks important literature on histomics and quantitative image phenotyping, which are highly relevant to the interpretation and biological validation of histology-based AI models.

Suggested references that should be discussed: "Phenotyping of Histology Imaging Data with Histomics",

"Radiomics in Medical Imaging: Methods, Applications, and Challenges"

These works provide important perspectives on quantitative tissue phenotyping, interpretable feature extraction, reproducibility, feature stability, and biological representation learning, all of which are directly relevant to the proposed endometrial histology analysis framework.

1. Limited Dataset Size Relative to Model Complexity

A major concern is the relatively small patient cohort used to train and evaluate extremely large deep learning models.

The training cohort consists of only 152 patients, while UNI2-h contains hundreds of millions of parameters. Although the feature extractor is frozen, the study still attempts to learn fertility-related representations from a limited number of cases.

The manuscript repeatedly attributes superior performance to foundation-model knowledge transfer, but it remains unclear whether observed improvements are statistically significant or merely reflect sampling variability.

The authors should discuss:

Dataset-size limitations more explicitly.

Risks of model instability and optimistic performance estimates.

Statistical power limitations.

Whether performance differences between models are statistically significant.

Confidence intervals alone are insufficient to demonstrate superiority.

2. External Validation Design is Problematic

The external cohort does not contain actual IVF outcome labels.

Instead, fertility status is inferred from menstrual phase timing:

LH+7 → labeled as successful

LH+3, LH+5, LH+9, LH+11 → labeled as unsuccessful

This introduces a substantial conceptual issue.

The internal model is trained to predict cumulative live birth outcomes, whereas external validation evaluates the ability to distinguish endometrial phases.

These are fundamentally different prediction targets.

Consequently, the reported external AUROC values may not truly represent generalization for live birth prediction.

The manuscript should explicitly acknowledge this limitation and avoid presenting external performance as a direct validation of IVF outcome prediction.

3. Insufficient Biological Interpretation

Although Grad-CAM visualizations are provided, biological interpretation remains superficial.

The study concludes that LE-associated regions contribute to prediction, but does not explain:

Which morphological characteristics drive predictions.

Whether attention corresponds to known implantation biology.

Whether glandular, stromal, vascular, inflammatory, or epithelial features are implicated.

The discussion remains largely model-centric rather than biology-centric.

More detailed pathological interpretation is necessary to support clinical relevance.

4. Missing Quantitative Histomics Perspective

The manuscript treats the problem entirely as a deep-learning classification task.

However, computational pathology increasingly emphasizes interpretable quantitative tissue phenotyping through histomics-based approaches.

The authors should discuss how their deep learning framework relates to established histomic descriptors such as:

Nuclear morphology

Cellular density

Glandular architecture

Spatial organization

Tissue texture patterns

The manuscript would be significantly strengthened by discussing findings in the context of:

Phenotyping of Histology Imaging Data with Histomics

This reference provides an important framework for linking computational image features with biologically meaningful tissue phenotypes.

5. Lack of Comparison with Handcrafted Feature Approaches

The study compares multiple deep learning architectures but does not include any traditional quantitative pathology baseline.

A comparison against:

Histomics features,

Morphological descriptors,

Classical machine learning models,

would provide valuable insight into whether foundation models truly outperform interpretable quantitative approaches.

Without such comparison, it is difficult to determine the added value of the proposed deep learning framework.

6. Interpretability Claims Are Overstated

The manuscript repeatedly emphasizes biological interpretability.

However, Grad-CAM alone does not provide mechanistic interpretability.

Grad-CAM merely highlights image regions associated with model decisions and does not explain:

Which features were learned,

Why predictions were made,

Whether highlighted regions are causally relevant.

The authors should moderate interpretability claims and acknowledge limitations of attention-based explanations.

7. No Assessment of Model Calibration

Clinical deployment requires more than discrimination metrics.

The study reports:

AUROC

Accuracy

Balanced accuracy

but does not evaluate calibration.

Important metrics such as:

Calibration curves

Brier score

Expected calibration error (ECE)

should be considered.

A model with good AUROC may still generate poorly calibrated probabilities unsuitable for clinical decision-making.

8. Limited Discussion of Reproducibility and Domain Shift

The study demonstrates substantial performance degradation of the HRT-trained model when applied to natural-cycle data.

This observation highlights a broader issue of domain shift.

However, the discussion does not sufficiently address:

Scanner variability

Staining variability

Institution-specific biases

Preprocessing variability

Batch effects

These challenges are central topics in quantitative imaging research.

The authors may benefit from discussing reproducibility principles described in:

Radiomics in Medical Imaging: Methods, Applications, and Challenges

which provides a comprehensive discussion of feature robustness, reproducibility, and external validation.

9. Statistical Analysis Needs Strengthening

The manuscript reports mean ± SD values across folds.

However, no statistical comparisons are presented between:

ResNet-18 vs UNI2-h

GAP vs ACMIL

Linear vs MLP

WSI vs LE models

Without statistical testing, claims regarding superiority remain largely descriptive.

Appropriate significance testing or confidence interval comparisons should be considered.

10. Clinical Utility Remains Unclear

Even the best-performing models achieve AUROCs around 0.84–0.90.

The manuscript does not explain:

How predictions would alter IVF decision-making.

Whether performance exceeds existing clinical assessment approaches.

Whether cost-benefit advantages exist.

How false-positive and false-negative predictions would affect patients.

The clinical translation pathway remains insufficiently developed.

**********

what does this mean?). If published, this will include your full peer review and any attached files.

**Do you want your identity to be public for this peer review?**  If you choose “no”, your identity will remain anonymous but your review may still be made public.

For information about this choice, including consent withdrawal, please see our Privacy Policy

Reviewer #1: No

Reviewer #2: No

Reviewer #3: No

**********

**Figure resubmission:**

**Reproducibility:**

To enhance the reproducibility of your results, we recommend that authors of applicable studies deposit laboratory protocols in protocols.io, where a protocol can be assigned its own identifier (DOI) such that it can be cited independently in the future. Additionally, PLOS ONE offers an option to publish peer-reviewed clinical study protocols. Read more information on sharing protocols at https://plos.org/protocols?utm_medium=editorial-email&utm_source=authorletters&utm_campaign=protocols

# Author comment: Author response to Decision Letter 1

**DOI:** 10.1371/journal.pdig.0001744.r002

5 Aug 2026

Attachment Submitted filename: Response_to_Reviewers_Endometrial_Histology_final.docx

# Peer review: Decision Letter 1

**DOI:** 10.1371/journal.pdig.0001744.r003

**Authors:** Feng Liu

27 Aug 2026

Response to ReviewersRevised Manuscript with Track ChangesManuscript**Journal Requirements:** **Additional Editor Comments (if provided):** **Reviewers' Comments:**

**Comments to the Author**

Reviewer #1: All comments have been addressed

Reviewer #2: (No Response)

**********

publication criteria?

Reviewer #1: Yes

Reviewer #2: Yes

**********

3. Has the statistical analysis been performed appropriately and rigorously?

Reviewer #1: N/A

Reviewer #2: Yes

**********

4. Have the authors made all data underlying the findings in their manuscript fully available (please refer to the Data Availability Statement at the start of the manuscript PDF file)?

The PLOS Data policy

Reviewer #1: Yes

Reviewer #2: No

**********

5. Is the manuscript presented in an intelligible fashion and written in standard English?

Reviewer #1: Yes

Reviewer #2: Yes

**********

**Review Comments to the Author**

Reviewer #1: Good work.

Reviewer #2: Thank you for the substantial revisions. The manuscript has improved considerably, and the authors have addressed the major methodological and statistical concerns raised in the previous round. In particular, the addition of formal DeLong testing with multiple-comparison adjustment, calibration assessment, clearer discussion of limited sample size and model uncertainty, improved treatment of technical domain shift, and more cautious interpretation of model comparisons have strengthened the study.

One point would benefit from further clarification before publication.

The external cohort is now appropriately described as an “external phase-based testing” cohort rather than a true external validation cohort for cumulative live birth. However, the Abstract still presents the internal and external AUROC values in parallel, which may give readers the impression that the external cohort directly validates live-birth prediction. Because the external cohort distinguishes LH+7 from non-LH+7 samples rather than using actual IVF/live-birth outcomes, I suggest making this distinction explicit in the Abstract at the point where the external performance results are reported. The conclusion should also continue to avoid implying that these external results constitute direct clinical validation of live-birth prediction.

Aside from this point, the authors have responded comprehensively to the previous reviewer concerns, and the revised manuscript is substantially stronger.

**********

what does this mean?). If published, this will include your full peer review and any attached files.

**Do you want your identity to be public for this peer review?**  If you choose “no”, your identity will remain anonymous but your review may still be made public.

For information about this choice, including consent withdrawal, please see our Privacy Policy

Reviewer #1: No

Reviewer #2: No

**********

**Figure resubmission:**

**Reproducibility:**

To enhance the reproducibility of your results, we recommend that authors of applicable studies deposit laboratory protocols in protocols.io, where a protocol can be assigned its own identifier (DOI) such that it can be cited independently in the future. Additionally, PLOS ONE offers an option to publish peer-reviewed clinical study protocols. Read more information on sharing protocols at https://plos.org/protocols?utm_medium=editorial-email&utm_source=authorletters&utm_campaign=protocols

# Author comment: Author response to Decision Letter 2

**DOI:** 10.1371/journal.pdig.0001744.r004

28 Aug 2026

Attachment Submitted filename: Response_to_Reviewers_PDIG-D-26-00729R2_minor_revision.docx

# Editor report: Decision Letter 2

**DOI:** 10.1371/journal.pdig.0001744.r005

**Authors:** Feng Liu

9 Sep 2026

Foundation model-powered deep learning of endometrial histology for predicting the cumulative live birth of an in vitro fertilization cycle

PDIG-D-26-00729R2

Dear Dr Lee,

We are pleased to inform you that your manuscript 'Foundation model-powered deep learning of endometrial histology for predicting the cumulative live birth of an in vitro fertilization cycle' has been provisionally accepted for publication in PLOS Digital Health.

Before your manuscript can be formally accepted you will need to complete some formatting changes, which you will receive in a follow-up email from a member of our team.

Please note that your manuscript will not be scheduled for publication until you have made the required changes, so a swift response is appreciated.

IMPORTANT: The editorial review process is now complete. PLOS will only permit corrections to spelling, formatting or significant scientific errors from this point onwards. Requests for major changes, or any which affect the scientific understanding of your work, will cause delays to the publication date of your manuscript.

If your institution or institutions have a press office, please notify them about your upcoming paper to help maximize its impact. If they'll be preparing press materials, please inform our press team as soon as possible -- no later than 48 hours after receiving the formal acceptance. Your manuscript will remain under strict press embargo until 2 pm Eastern Time on the date of publication. For more information, please contact digitalhealth@plos.org.

Thank you again for supporting Open Access publishing; we are looking forward to publishing your work in PLOS Digital Health.

Best regards,

Feng Liu, Ph.D.

Section Editor

PLOS Digital Health

***********************************************************

**Additional Editor Comments (if provided):**

**Reviewer Comments (if any, and for reference):**
